# The Impact of Technology-Enabled Care Coordination in a Complex Mental Health System: A Local System Dynamics Model

**DOI:** 10.2196/25331

**Published:** 2021-06-30

**Authors:** Frank Iorfino, Jo-An Occhipinti, Adam Skinner, Tracey Davenport, Shelley Rowe, Ante Prodan, Julie Sturgess, Ian B Hickie

**Affiliations:** 1 Brain and Mind Centre University of Sydney Sydney Australia; 2 Translational Health Research Institute Western Sydney University Sydney Australia; 3 North Coast Primary Health Network Coffs Harbour Australia

**Keywords:** medical informatics, internet, care coordination, complex systems, simulation, health systems, policy, mental health

## Abstract

**Background:**

Prior to the COVID-19 pandemic, major shortcomings in the way mental health care systems were organized were impairing the delivery of effective care. The mental health impacts of the pandemic, the recession, and the resulting social dislocation will depend on the extent to which care systems will become overwhelmed and on the strategic investments made across the system to effectively respond.

**Objective:**

This study aimed to explore the impact of strengthening the mental health system through technology-enabled care coordination on mental health and suicide outcomes.

**Methods:**

A system dynamics model for the regional population catchment of North Coast New South Wales, Australia, was developed that incorporated defined pathways from social determinants of mental health to psychological distress, mental health care, and suicidal behavior. The model reproduced historic time series data across a range of outcomes and was used to evaluate the relative impact of a set of scenarios on attempted suicide (ie, self-harm hospitalizations), suicide deaths, mental health–related emergency department (ED) presentations, and psychological distress over the period from 2021 to 2030. These scenarios include (1) business as usual, (2) increase in service capacity growth rate by 20%, (3) standard telehealth, and (4) technology-enabled care coordination. Each scenario was tested using both pre– and post–COVID-19 social and economic conditions.

**Results:**

Technology-enabled care coordination was forecast to deliver a reduction in self-harm hospitalizations and suicide deaths by 6.71% (95% interval 5.63%-7.87%), mental health–related ED presentations by 10.33% (95% interval 8.58%-12.19%), and the prevalence of high psychological distress by 1.76 percentage points (95% interval 1.35-2.32 percentage points). Scenario testing demonstrated that increasing service capacity growth rate by 20% or standard telehealth had substantially lower impacts. This pattern of results was replicated under post–COVID-19 conditions with technology-enabled care coordination being the only tested scenario, which was forecast to reduce the negative impact of the pandemic on mental health and suicide.

**Conclusions:**

The use of technology-enabled care coordination is likely to improve mental health and suicide outcomes. The substantially lower effectiveness of targeting individual components of the mental health system (ie, increasing service capacity growth rate by 20% or standard telehealth) reiterates that strengthening the whole system has the greatest impact on patient outcomes. Investments into more of the same types of programs and services alone will not be enough to improve outcomes; instead, new models of care and the digital infrastructure to support them and their integration are needed.

## Introduction

Mental illness is a major cause of disability and early mortality globally [1-3]. The early onset, chronicity, and complexity of mental illness means that the human, social, and economic costs are enormous [4]. Yet, mental health systems often struggle to provide adequate care that effectively intervenes to mitigate the short- and long-term negative outcomes [5].

For some, access to mental health care has improved [6-9]; however, most health systems lack effective coordination between service silos, which impacts on the delivery of holistic, timely, and quality mental health care [10,11]. Service fragmentation, delayed care, mental health treatment isolated from other physical and social needs, complicated service pathways, and inefficient resource allocation are persistent features of an underperforming service system [5,12]. This is particularly problematic for people with more complex needs who tend to report higher rates of disengagement from education or employment [7-9], suicidality [13,14], and comorbidity [15]. These cases are the norm among those who frequently engage with health systems and typically require multidisciplinary team-based care approaches [16]. These approaches embrace collaborative care models, which recognize that effective care coordination between service providers, including intensive assessment, personalized treatment plans, targeted referrals, clinical information systems use, and outcome monitoring can improve treatment engagement, satisfaction with care, and mental health outcomes [17-19].

The integrated use of digital technologies offers significant potential to enable effective coordination of mental health care [20-23]. The accessibility, scalability, and standardization mean that technology is well-placed to play a major role in the digitization of health care and can be leveraged to deliver quality mental health care across settings, especially those that are remote or rural and may be underresourced when compared to urban centers [24]. Their use in mental health systems has already demonstrated utility to improve access to care and communication between service providers and consumers [25]. This may be particularly important for people with more complex needs, such as housing and employment support, whereby mental health treatment may be an additional burden in terms of time, effort, and finance. Technology offers an opportunity to alleviate some of this burden through greater flexibility in terms of access to effective care, greater efficiencies through the sharing of information between providers, and improving engagement with care [26].

Never has this capability been more vital than amid a pandemic and recession whose effects are disrupting nearly every aspect of life: familial, educational, vocational, health, and social structures. This disruption is threatening population mental health and well-being and is likely to generate service demand of unprecedented magnitude for many years [27,28]. Some governments are responding by instituting measures to reduce economic and social hardship, investing in mental health programs and services, and improving access via virtual mental health services (ie, basic telehealth). These investments represent a move in the right direction; however, without addressing the fundamental models of care, the rapid adoption of telehealth poses the risk that it may digitize the problems that already exist in existing models of care across the mental health system [29,30].

Systems modeling and simulation is a low-risk method of exploring likely impacts of counterfactual scenarios. This study uses an existing local system dynamics model developed for regional mental health services planning to identify the impact of using digital technologies to facilitate care coordination on mental health outcomes and health system burden (ie, technology-enabled care coordination). Here, we aimed to compare technology-enabled care coordination to three other likely intervention scenarios: (1) business as usual, (2) an increase in the growth rate of existing service capacity (ie, increasing throughput of people into existing systems), and (3) digital technologies used to extend existing services online (eg, via videoconferencing) without changing the underlying model of care (ie, standard telehealth). We aimed to model the impacts of each scenario within both a *typical* context and a public mental health crisis resulting from the pandemic and economic recession.

## Methods

### Context

The North Coast NSW (New South Wales) Primary Health Network supports a population of 502,524, as of 2016 [31], distributed over a geographic area of approximately 35,570 square kilometers and takes in both coastal and inland rural communities. The region is more socioeconomically disadvantaged compared to the state and national averages, with higher rates of unemployment, domestic and family violence, and homelessness [32,33].

### Model Overview

The system dynamics model was developed using a participatory modeling approach that involved over 50 local stakeholders, including representatives from health and social policy agencies, local government, nongovernmental organizations, primary care providers, emergency services, research institutions, community groups, and, importantly, people with lived experience. The process employed a broad systems perspective drawing on the deep tacit knowledge of the diverse perspectives of these system actors. Input from stakeholders was provided through a series of workshops, meetings, surveys, and local system mapping activities between July and December 2019. This process involved iteratively working on the model structure and assumptions; regular face validity checks by a diverse group of academic, clinical, policy, program planning, emergency services, and lived-experience stakeholders were undertaken to ensure accurate model representation, conceptualization, and outputs. A more detailed description of the model development process, structure, outputs, and calibration can be found in the primary paper [34].

In summary, the core model structure included the following dynamically interacting components:

1. A population component, capturing changes over time in the size and structure of the population resulting from births, net migration, and mortality across the following age groups: 0-14 years, 15-24 years, 25-64 years, and 65+ years old.
2. A psychological distress component that captures changes in the rates and severity of psychological distress in the population (ie, states of low psychological distress, with a Kessler 10 [K10] score of 10-15; moderate psychological distress, with a K10 score of 16-21; and high to very high psychological distress, with a K10 score of 22-50).
3. A series of components capturing pathways within and between key social determinants of mental health and suicidal behavior, namely, early life exposures, substance abuse, domestic violence, homelessness, and unemployment.
4. A mental health service system component that models the movement of psychologically distressed people through one of several possible service pathways, potentially involving general practitioners; psychiatrists; allied mental health professionals, including psychologists and mental health nurses; emergency department (ED) and psychiatric inpatient care; community- and hospital-based outpatient care; and online services, and one that captures changes over time in service demand and capacity.
5. A suicidal behavior component that captures self-harm hospitalizations and suicide deaths. Figure S1 in Multimedia Appendix 1 details the key social determinants identified by the participatory process and their hypothesized impact on mental health and the other model outcomes.

Model construction and analysis were performed using Stella Architect, version 1.9.4 (isee systems inc). The model was validated by (1) testing whether the model could replicate historic data across a range of key indicators, namely, time series of psychological distress, psychiatric hospitalizations, mental health–related ED presentations, self-harm hospitalizations, and suicide deaths, and (2) ensuring face validity among diverse system actors in the model structure and performance. The AdViSHE (Assessment of the Validation Status of Health-Economic decision models) checklist was also used to assess the validation status of this model and its outputs [35].

### Model Outputs

For this study, model outputs included both mental health outcomes and service usage for the total population. Mental health outcomes and health system burden include total cumulative numbers of self-harm hospitalizations, which are a proxy for suicide attempts; suicide deaths; prevalence of high to very high psychological distress; and mental health–related ED hospitalizations. All outputs were calculated every 0.4375 days (ie, one-sixteenth of a week) starting from January 1, 2011; these permitted comparisons of model outputs with historic data from 2011 to 2017 for validation. Forecasts of the impacts of intervention scenarios described below are simulated from the time of implementation in 2021 to the end of 2029 [34].

### Intervention Scenarios

Four distinct intervention scenarios were tested. The first scenario is *business as usual*, whereby there is no change to the existing services system and the rate of growth in existing services is maintained. The second scenario is *an increase in service capacity growth by 20%*, whereby the yearly growth of existing service capacity is increased. These services include those provided by general practitioners; psychiatrists; allied mental health professionals, including psychologists and mental health nurses; and community-based mental health services. The third scenario is *standard telehealth*, whereby digital technologies are used to extend existing services online (eg, via videoconferencing) without changing the underlying model of care. The fundamental assumption here is that technologies are being used to remotely provide existing models of care delivered by individual providers rather than being used to improve the coordination of care between service providers: a coordinated, multidisciplinary, team-based approach to care. This means that there is no change to referrals between services nor to the per-service recovery rate, since the only element that has changed is the mode of delivery. The fourth scenario is *technology-enabled care coordination*, which involves the use of online technology to facilitate delivery of multidisciplinary team-based care, in which medical and allied health professionals consider all relevant treatment options and collaboratively develop an individual treatment and care plan for each patient. Online technology enables enhanced coordination of care and facilitates communication between medical and allied health professionals, since each health professional involved in the care of a patient has access to the same information about that patient’s treatment history (see parameter details in Textbox 1 [17,25,34,36,37]).

Each scenario was operationalized by varying key parameter estimates in the model, as outlined in Table 1. Each of these intervention scenarios was tested under *pre–COVID-19 conditions*, which are based on original trends in socioeconomic circumstances (ie, all policies and programs currently in place remain unchanged and service capacity continues to increase at current rates). Each intervention scenario was also tested under *post–COVID-19 conditions*, which reflect a new baseline for socioeconomic circumstances to compare and contrast the qualitative and quantitative performance of this intervention when faced with new realities (eg, socioeconomic shocks). The interventions considered here are not intended to be a complete evaluation of strategies to mitigate the effects of the COVID-19 pandemic, but rather we aimed to determine the robustness of each scenario under these changed conditions. Table 2 details how the pre– and post–COVID-19 initial conditions were implemented in the model.

**Parameters determining the direct effects of technology-enabled care coordination [34].**

*Maximum rate per service:*

This refers to the maximum proportion of mental health services provided that involve technology-enabled care coordination. This proportion will depend on the number of medical and allied mental health professionals adopting technology-enabled care coordination, as well as the number of patients consenting to the use of these technologies in the management of their care (ie, uptake among service providers and patients). The default value (0.7) assumes that when fully implemented, technology-enabled care coordination will be provided in 70% of mental health services completed.

*Effect on recovery rate:*

This refers to the multiplicative effect of technology-enabled care coordination on the per-service recovery rate (ie, the probability that a patient’s level of psychological distress will decrease after receiving treatment). The default estimate (1.177) implies that technology-enabled care coordination increases the per-service probability of a reduction in psychological distress by 17.7% [17,25].

*Effect on referrals to specialized care:*

This refers to the multiplicative effect of technology-enabled care coordination on general practitioners’ rates of referral to specialized psychiatric care (ie, psychiatrists and allied mental health services). The default value (1.266) implies that technology-enabled care coordination increases the per-consultation probability that a general practitioner will refer a patient with high or very high psychological distress to specialized psychiatric care by 26.6% [36]. Note that technology-enabled care coordination is assumed to have no effect on the referral rate for patients with moderate psychological distress.

*Effect on disengagement:*

This refers to the multiplicative effect of technology-enabled care coordination on rates of disengagement from mental health services, including waiting for services and dissatisfaction with services received. The default estimate (0.520) implies that technology-enabled care coordination reduces rates of disengagement by 48.0% [25,36].

*Effect on referrals to alcohol and other drugs services:*

This refers to the multiplicative effect of technology-enabled care coordination on the rate of referral of patients with a substance abuse disorder to alcohol and drug treatment services. The default value (1.1) assumes an increase in the rate of referral of 10% (ie, patients with a substance abuse disorder receiving technology-enabled care coordination are 10% more likely to be referred to alcohol and drug treatment services than patients with a substance abuse disorder receiving usual care).

*Effect on substance use relapse rate:*

This refers to the multiplicative effect of coordinated treatment of co-occurring substance abuse and mental disorders on the substance use relapse rate (ie, the probability that a patient treated for a substance use disorder will relapse when treatment is completed). The default value (0.869) implies that coordinated substance abuse and psychiatric treatment reduces the rate of substance use relapse by 13.1% (ie, compared to substance abuse treatment alone) [37].

*Effect on employment initiation:*

This refers to the multiplicative effect of technology-enabled care coordination on the rate at which unemployed patients gain employment, through referral to employment services. The default value (1.1) assumes an increase in the employment initiation rate of 10% (ie, unemployed patients receiving technology-enabled care coordination are 10% more likely to gain employment than unemployed patients receiving usual care).

*Effect on exiting homelessness rate:*

This refers to the multiplicative effect of technology-enabled care coordination on the rate at which homeless patients secure housing, through referral to homelessness services. The default value (1.1) assumes a 10% increase in the rate that patients exit homelessness, equal to the inverse of the duration of homelessness (ie, homeless patients receiving technology-enabled care coordination are 10% more likely to secure housing than homeless patients receiving usual care).

*Effect on psychiatric service capacity:*

This refers to the multiplicative effect of technology-enabled care coordination on the total capacity of specialized psychiatric services (ie, the maximum number of services that can be provided by psychiatrists and allied mental health providers per year). The default value (1.1) assumes an increase in service capacity of 10%.

*Effect on referrals to online services:*

This refers to the multiplicative effect of technology-enabled care coordination on the rate of referral of patients with moderate psychological distress to online services. The default value (1.1) assumes an increase in the rate of referral of 10% (ie, patients with moderate psychological distress receiving technology-enabled care coordination are 10% more likely to be referred to online services than patients with moderate psychological distress receiving usual care).

**Table 1:** Parameter values for each intervention scenario.

Parameter^a^			Parameter values for each scenario						
			Scenario 1^b^		Scenario 2^c^		Scenario 3^d^		Scenario 4^e^
**Direct effects on technology interventions**									
	Maximum rate per service	0		0		0.70		0.70	
	Effect on recovery rate	1.00		1.00		1.09		1.18	
	Effect on referrals to specialized care	1.00		1.00		10.00		1.27	
	Effect on disengagement	1.00		1.00		0.76		0.52	
	Effect on alcohol and other drugs services referral rate	1.00		1.00		1.00		1.10	
	Effect on substance abuse relapse	1.00		1.00		0.93		0.87	
	Effect on employment	1.00		1.00		1.00		1.10	
	Effect on exiting homelessness rate	1.00		1.00		1.00		1.10	
	Effect on service capacity	1.00		1.00		1.10		1.10	
	Effect on referrals to online services	1.00		1.00		1.00		1.10	
**Direct effects on service capacity**									
	General practice service capacity increase per year	125.85		151.02		125.85		125.85	
	Psychiatrist and allied service capacity increase per year	216.31		259.57		216.31		216.31	
	CMHC^f^ service capacity increase per year	0		75.14		0		0	

^a^See Textbox 1 for more details about each parameter.

^b^Scenario 1: business as usual.

^c^Scenario 2: increase in service capacity growth rate by 20%.

^d^Scenario 3: standard telehealth.

^e^Scenario 4: technology-enabled care coordination.

^f^CMHC: community mental health capacity.

**Table 2:** Comparison of parameters used to model pre– and post–COVID-19 initial conditions.

Parameter	Parameter values for each condition	
	Pre–COVID-19	Post–COVID-19
Youth job loss rate ratio	1.00	5.00
Unemployment total	1.00	15.00
Unemployment effect decay rate	0	0.05
Sense of community index (ie, social connectedness)	9.61	8.24
Years to reach sense of community index	0	1.00
Duration (years) of social disconnection	0	2.00

### Statistical Analysis

Sensitivity analyses were performed to assess the impact of uncertainty in parameter estimates of the direct effects of each intervention scenario on the simulation results. Latin hypercube sampling was used to draw 100 sets of values for selected model parameters determining the direct effects of the interventions on suicidal behavior in young people, from a uniform joint distribution spanning ±20% of the default values. Differences in each of the model outputs (eg, cumulative numbers of self-harm hospitalizations, suicide deaths, and mental health–related ED presentations) between the baseline and intervention scenarios were calculated for each set of parameter values and were summarized using simple descriptive statistics. All intervals reported in this paper are derived from the distributions of model outputs calculated in the sensitivity analyses; they provide a measure of the impact of uncertainty in the intervention effect estimates but should not be interpreted as confidence intervals.

## Results

### Overview

**Figure 1:**
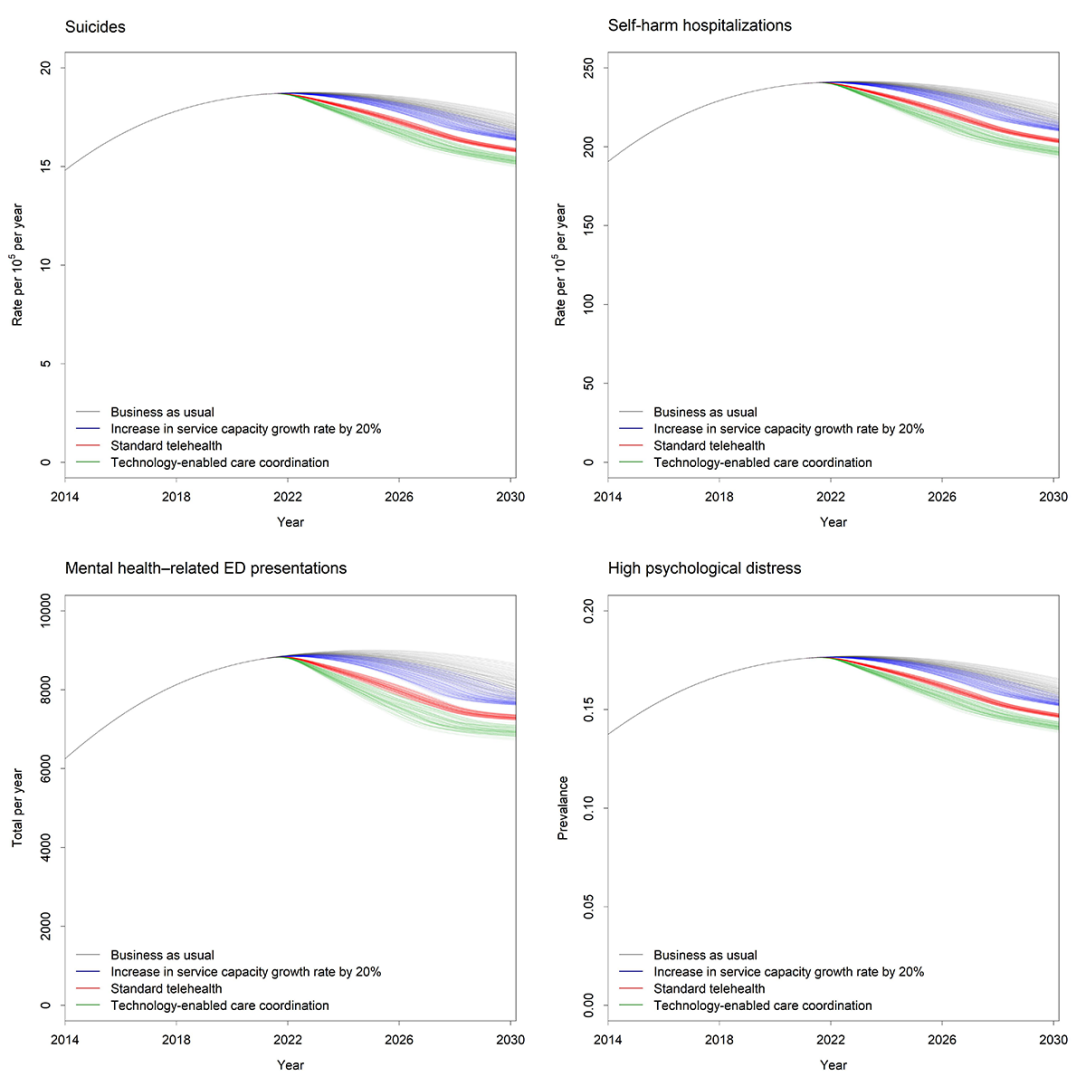
Mental health outcomes for each intervention scenario. ED: emergency department.

**Figure 2:**
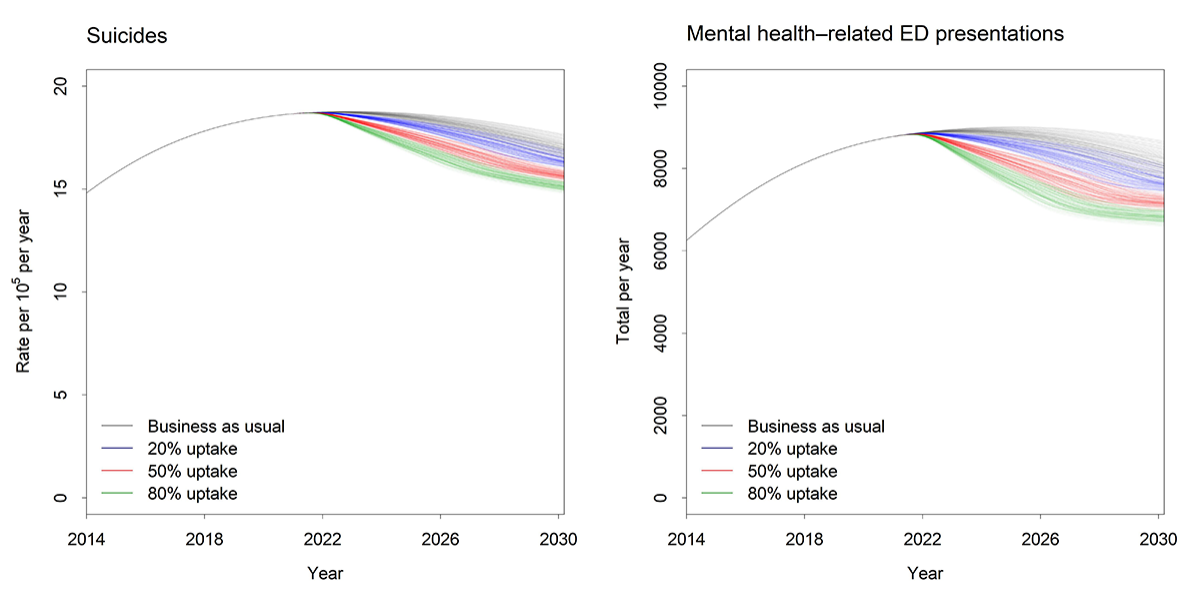
The impact of the rate of uptake on mental health outcomes. ED: emergency department.

Under the baseline scenario (ie, business as usual pre–COVID-19), approximately 12,274 self-harm hospitalizations, 953 suicide deaths, and 81,263 mental health–related ED presentations were forecast for the period from 2021 to 2030. By 2030, the prevalence of high psychological distress was expected to be on a trajectory of decline and was set to reduce by 2 percentage points (ie, from 17.60% in 2021 to 15.60% in 2030). Figure 1 shows the relative impact of each scenario on mental health and suicide outcomes. The implementation of technology-enabled care coordination (scenario 4) had the largest impact on outcomes for the total population. For the total population, self-harm hospitalizations and suicide deaths were reduced by 6.71% (95% interval 5.63%-7.87%), mental health–related ED presentations were reduced by 10.33% (95% interval 8.58%-12.19%), and the prevalence of high psychological distress in 2030 was reduced by 1.76 percentage points (95% interval 1.35-2.32 percentage points). This would result in the prevention of 844 self-harm hospitalizations, 66 suicides, and 8448 mental health–related hospitalizations over the forecast period and a decline in the prevalence of high psychological distress to 14% in 2030. The rate of uptake of technology-enabled care coordination has a major impact on the effects of this intervention (Figure 2). The impact on all outcomes is greater as the rate of uptake across the mental health system increases from 20% to 50% and then to 80%.

Increasing the service capacity growth rate by 20% (scenario 2) only had a minor impact on both populations; for the total population, it was forecast to reduce self-harm hospitalizations and suicide deaths by 1.64% (95% interval –0.20% to 3.51%), mental health–related ED presentations by 2.76% (95% interval –0.41% to 5.95%), and the prevalence of high psychological distress in 2030 by 0.57 percentage points (95% interval –0.08 to 1.29 percentage points). This would result in the prevention of 274 self-harm hospitalizations, 21 suicides, and 3023 mental health–related hospitalizations. The prevalence of high psychological distress was projected to decline to 15% in 2030.

When digital technologies were used for standard telehealth (scenario 3), the impact on outcomes was better than scenario 2 but lower than scenario 4, reducing self-harm hospitalizations and suicide deaths by 3.50% (95% interval 1.58%-5.03%), mental health–related ED presentations by 5.48% (95% interval 2.24%-5.43%), and the prevalence of high psychological distress in 2030 by 0.99 percentage points (95% interval 0.42-1.67 percentage points). Over the forecast period, this would result in the prevention of 418 self-harm hospitalizations, 32 suicides, 4244 mental health–related hospitalizations, and a decline in the prevalence of high psychological distress to 14.8% in 2030.

### Post–COVID-19 Scenario Modeling

The impact of the public mental health crisis was estimated to have detrimental effects on all outcomes, with 14,973 self-harm hospitalizations (22% increase from baseline figures), 1176 suicide deaths (23% increase), and 98,591 mental health–related ED presentations (21% increase). By 2030, the prevalence of high psychological distress was forecast to be 18.3%, a 2.7–percentage point increase from the baseline figures forecast for 2030. During this public mental health crisis, technology-enabled care coordination (scenario 4) was forecast to have a similar impact on outcomes, with a 6.43% (95% interval 5.29%-7.46%) reduction in self-harm hospitalizations, a 6.40% (95% interval 5.26%-7.42%) reduction in suicide deaths, a 10.01% (95% interval 8.06%-11.71%) reduction in mental health–related ED presentations, and a reduction of 2.52 percentage points (95% interval 1.82-3.08 percentage points) in high psychological distress in 2030. The pattern of results for each scenario was consistent under these changed conditions; technology-enabled care coordination represents an important and effective part of the strategy to mitigate the social and economic impacts of the COVID-19 pandemic, with outcomes reduced back down to the initial *business as usual* estimates from pre–COVID-19.

## Discussion

### Principal Findings

These findings suggest that the use of technology-enabled care coordination is likely to result in better mental health outcomes and reduce health system burden at a population level. When compared to increasing the service capacity growth rate across a variety of settings by 20% and standard telehealth, technology-enabled care coordination led to greater reductions in suicide deaths, the total number of self-harm hospitalizations or mental health–related ED hospitalizations, and the prevalence of high psychological distress in the population. This emphasizes that strengthening how the whole mental health system functions together will have a greater impact on outcomes than simply improving the capacity across individual components of the mental health system. Investments into more of the same types of programs and services alone will not be enough to improve the outcomes for the whole system; instead, new models of care and the infrastructure to support them and their integration need to accompany these investments.

Increasing the service capacity growth rate does have a minor impact; however, since it primarily acts to increase throughput into the current mental health system, it does not address existing challenges of service fragmentation. Many structural barriers and misaligned incentives remain across the system, which contribute to the health system burden and inefficient allocation of resources that often result in poor outcomes for individuals [38-40]. Addressing these systemic issues will most likely involve ensuring that the mental health system has the appropriate infrastructure in place to not only meet the demand for services, but provide timely care that appropriately targets the diverse needs of people presenting for care.

This work supports calls for increased use of digital technologies in mental health care [41]; however, it also suggests the importance of the way these technologies are employed. The modeling shows that when digital technologies are used for standard telehealth practices by extending existing services online (eg, via videoconferencing), without changing the underlying model of care, then the impact is lower. This type of scenario reflects what we might expect to see when telehealth is more widely implemented to deliver existing services, yet little effort is made to utilize these technologies in ways that promote multidimensional team-based care and maximize the benefits that technologies provide. As such, while telehealth stands to improve access, reach, and throughput to the mental health system and is a critical improvement in health service delivery, it does not necessarily ensure that people receive the appropriate type of mental health and social care that improves their chances of recovery. For this, a transformation in the models of care provided by services within the mental health system are required to achieve the full potential benefits of digital technologies.

Research regarding the effectiveness of digital technologies for mental health is growing, yet many of these innovations focus on the use of technologies within closed systems of care, often in isolation from other parts of the mental health system [30,42]. This neglects the way people typically need to access multiple parts of the system to receive effective care. This study illustrates that the benefits of technology-enabled care coordination for the whole system continues to increase as the proportion of services using this intervention increases. Unfortunately, realizing this type of widespread usage will require overcoming specific implementation barriers that have plagued most attempts to implement new technologies into existing health systems [22,43]. Common barriers include technology design, variation in the level of integration into existing service pathways and clinical protocols, process dynamics, contextual factors (eg, local leadership and organizational support), and other factors (eg, resourcing and training) [44-46]. Addressing these barriers requires a whole system approach that challenges the traditional and often rigid health systems to ensure that these tools are developed and integrated with services in a way that truly transforms clinical practice for the whole system.

This work should be considered in light of some limitations. The use of multiple secondary data sets introduces potential measurement bias for the estimates used to parameterize the model. Strategies were used to reduce the impact of such biases, including the triangulation of multiple data sources, parameter estimation via constrained optimization, and local verification to identify plausible estimates. The impacts of simulated scenarios are not necessarily generalizable to other regions, due to the specificity of the population, demographic, behavioral, social, economic, and mental health service dynamics that drive outcomes in a particular modeled region. Yet, for regions contextually similar to the modeled region, many of the model insights are likely to be relevant and provide a compelling case for exploring the likely impacts of similar technologies elsewhere. Further, the distinctions between the assumptions of scenarios 3 and 4 are likely to provide more generalizable insights about what components of digital transformation are projected to have large impacts in health systems. Future work should also focus on validating these model outputs over time by embedding them in local monitoring and evaluation of the implemented technology. In addition, comparisons of the modeled impacts of the technology applied to regions with similar or different social, economic, and mental health system contexts will provide additional broader insights. Finally, this study focuses on population effects of scenarios whereby the effects of interventions are generalized at the population level and cannot account for individual differences in the way that digital technologies may be rolled out or implemented at a local level. To address these specific issues, individual-level approaches, such as agent-based modeling, may be more appropriate to determine the effect of digital technologies on different agents within a mental health system (eg, people, clinicians, and services) and the impact this has on mental health and suicide outcomes.

### Conclusions

Systems modeling and simulation of the likely impact of technology-enabled care coordination in ordinary and extraordinary times has highlighted its significant potential in improving population mental health and suicide outcomes. This work also provides important evidence to support a push for major investment to scale up the implementation of digital technologies that support new models of care facilitating care coordination.

## Appendix

Multimedia Appendix 1 Supplementary materials.

## Abbreviations

AdViSHE: Assessment of the Validation Status of Health-Economic decision models
BMC: Brain and Mind Centre
ED: emergency department
K10: Kessler 10
NSW: New South Wales
