# Supplementary material for: The Impact of Technology-Enabled Care Coordination in a Complex Mental Health System: A Local System Dynamics Model
Source: J Med Internet Res. 2021 Jun 30;23(6):e25331. doi: 10.2196/25331 (PMC8274674; doi:10.2196/25331)
Supplement: Multimedia Appendix 1 [file jmir_v23i6e25331_app1.docx]

**Supplementary material**

1. Model structure

*1.1. Overview*

The system dynamics model used for the simulation experiments presented in the paper comprises a set of interconnected sub-models, or sectors, that includes: 1) a population sector, capturing changes in population size and structure resulting from births, migration, aging, and mortality; 2) a psychological distress sector that models flows of people to and from states of low, moderate, and high to very high psychological distress; 3) an early life sector modelling exposure to childhood adversity and its effect on the risk of developing mental disorders in adolescence and adulthood; 4) an employment sector, capturing changes in labour force status in the working-age population (15 years and above); 5) a substance abuse sector that captures the incidence of substance abuse disorders and the flow of patients through alcohol and other drug (AOD) treatment services; 6) domestic violence and homelessness sectors that model the age-specific incidence of intimate partner violence and transitions into and out of homelessness; 7) a mental health services sector, modelling the movement of distressed patients through a network of possible service pathways involving (potentially) general practitioners, community-based mental health services (including psychiatrists, psychologists and other allied health providers, and hospital outpatient services), emergency departments, general and psychiatric hospital inpatient care, and online services; and 4) a suicidal behaviour sector that captures numbers of self-harm hospitalisations and suicides. Figure S1 presents a high-level map of the core model showing the (causal) connections among sectors.

*1.2. Population sector*

Figure S2 shows the structure of the population sector, which captures changes in the size and composition of the northern New South Wales (NSW) population resulting from births, migration, aging, and mortality. The total population of the North Coast Primary Health Network (PHN) catchment is represented as four stocks (i.e., state variables), corresponding to the numbers of people aged 0−14 years, 15−24 years, 25−64 years, and 65 years and above. Population size increases through births (which are added to the stock of 0−14-year-olds) and immigration and decreases via emigration and mortality. Aging is modelled as a first-order delay, in which people flow out of each stock (except the stock of people aged ≥ 65 years) at a rate $n/d$, where $n$ is the number of people in the stock at any particular time point, and the average delay time $d$ is the mean number of years a person spends in the stock. Births and deaths occur at rates $bP$ and $\theta_{i}mP_{i}$, respectively, where $P$ is the total population, $\theta_{i}$ and $P_{i}$ are, respectively, the mortality rate ratio and population for age group $i$, and the per capita birth rate $b$ and per capita mortality rate for the total population $m$ decline at constant fractional rates per year. Net migration for age group $i$ is equal to $I_{i}-e_{i}P_{i}$, where $I_{i}$ is total age-specific immigration per year and $e_{i}$ is the age-specific per capita emigration rate per year. Population estimates derived from the system dynamics model are presented alongside estimates from HealthStats NSW (http://www.healthstats.nsw.gov.au/) in figure S3.


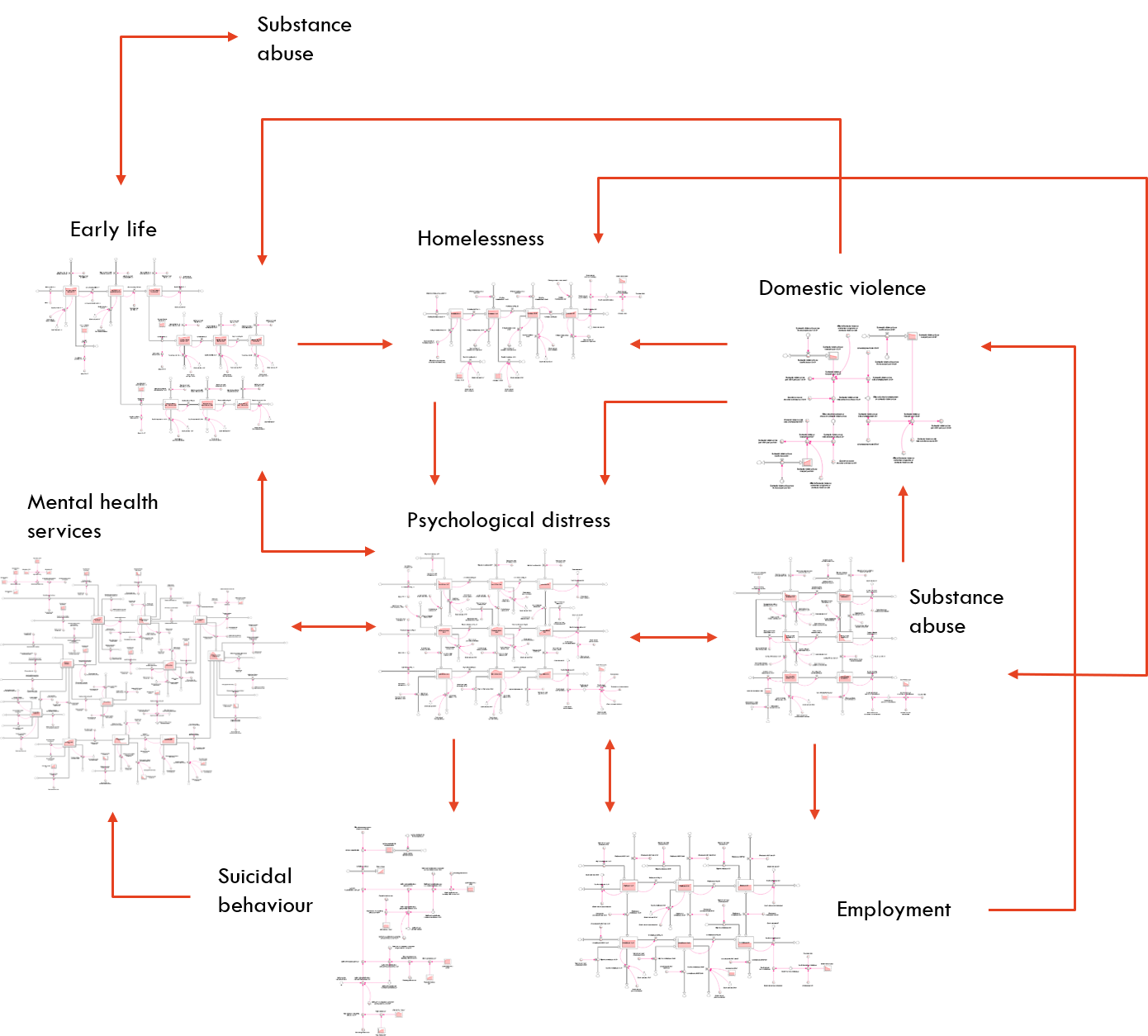


Figure S1. High-level map of the core system dynamics model showing the causal connections among model sectors. Single-headed arrows indicate unidirectional causal connections; bidirectional causal connections are shown as double-headed arrows.


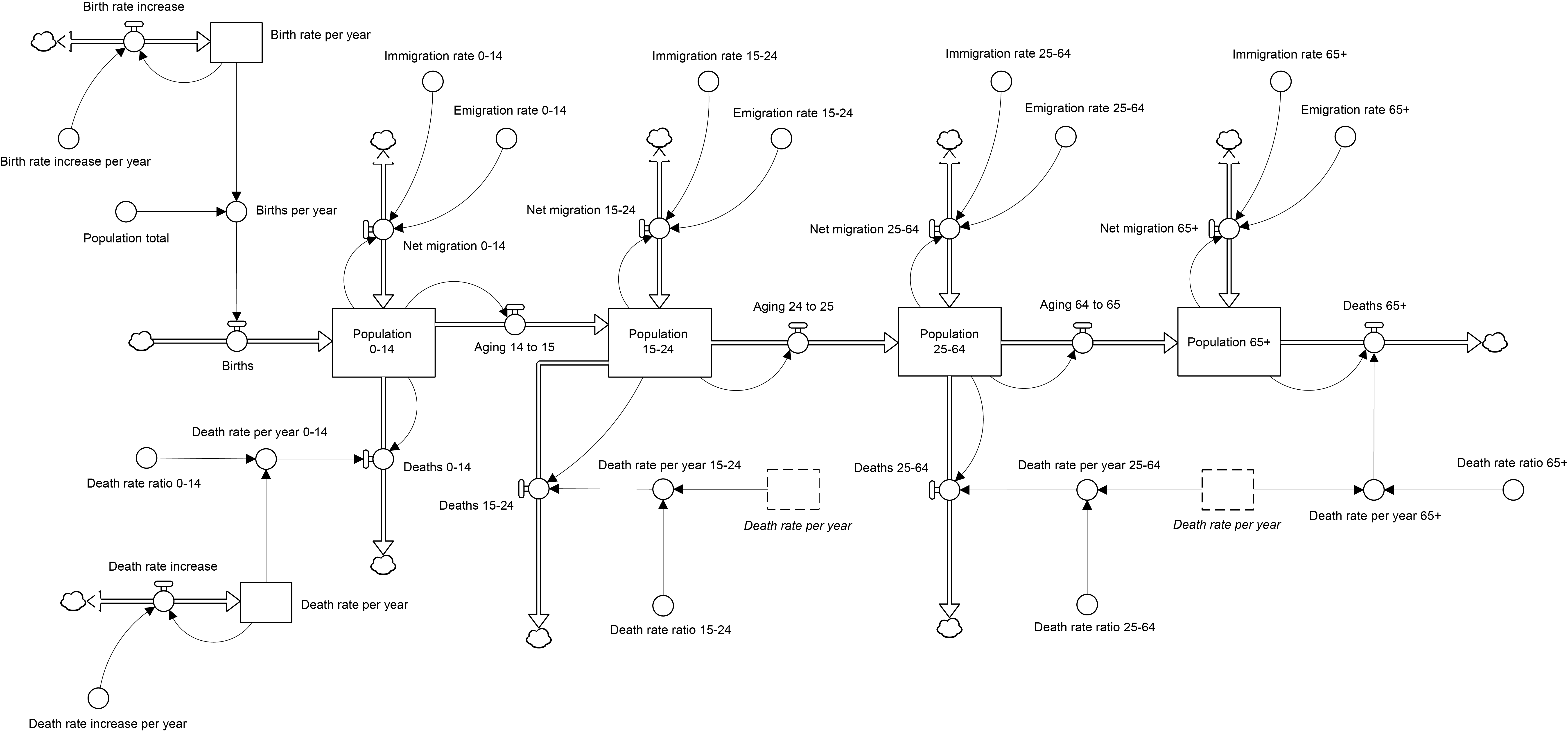


Figure S2. Structure of the population sector.


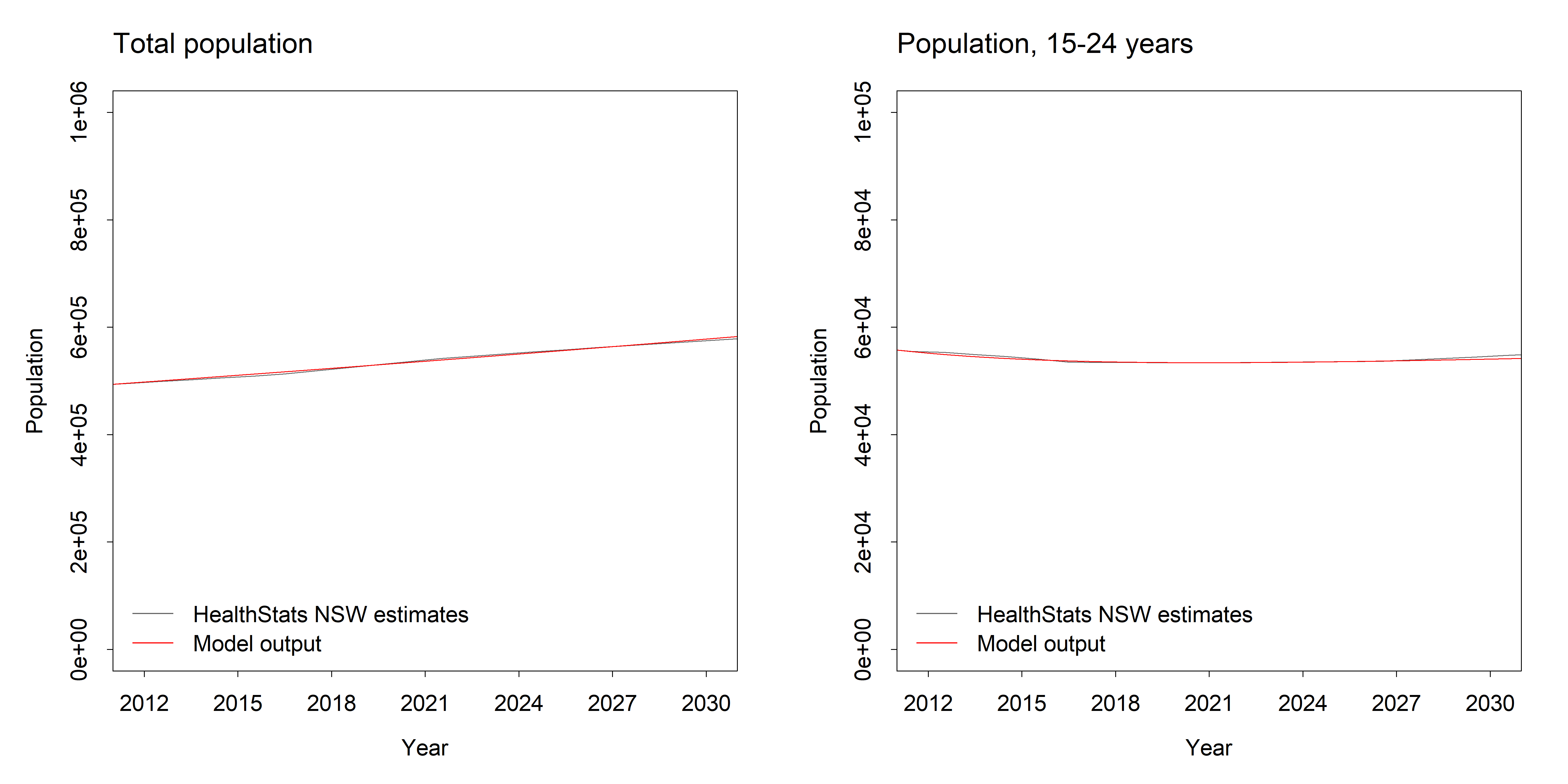


Figure S3. Population estimates (all ages and 15−24-year-olds) derived from the system dynamics model and from HealthStats NSW (http://www.healthstats.nsw.gov.au/).

*1.3. Psychological distress sector*

The psychological distress sector captures transitions between states of low psychological distress (Kessler 10 [K10] scores 10−15), moderate psychological distress (K10 scores 16−21), and high to very psychological distress (K10 scores above 21) within each age group (except 0−14-year-olds; figure S4). Adolescents flow into stocks of people aged 15−24 years with low, moderate, and high to very high psychological distress at rates equal to ${R_{l}}/{d_{l}}$, ${R_{m}}/{d_{m}}$, and ${R_{h}}/{d_{h}}$, respectively, where $R_{l}$, $R_{m}$, and $R_{h}$ are the numbers of 0−14-year-olds at low, moderate, and high risk of developing mental disorders, respectively, and $d_{l}$, $d_{m}$, and $d_{h}$ are the corresponding mean lengths of time children spend in each risk state before turning 15 years (see section 1.4). Aging of people experiencing low, moderate, or high to very high psychological distress is modelled using the same approach described for the population sector above; i.e., as a first-order delay (see section 1.2). Transitions are assumed to occur only between adjacent distress states, so that people with low psychological distress can become moderately distressed, but do not transition directly to a state of high to very high distress (although they may become highly distressed after becoming moderately distressed); similarly, people experiencing high to very high psychological distress can only transition directly to a state of moderate distress.

Net flows from lower to higher levels of psychological distress (i.e., from low to moderate distress, and from moderate to high or very high distress) are calculated as $hsL-\left( rH+T \right)$, where $L$ and $H$ are the numbers of


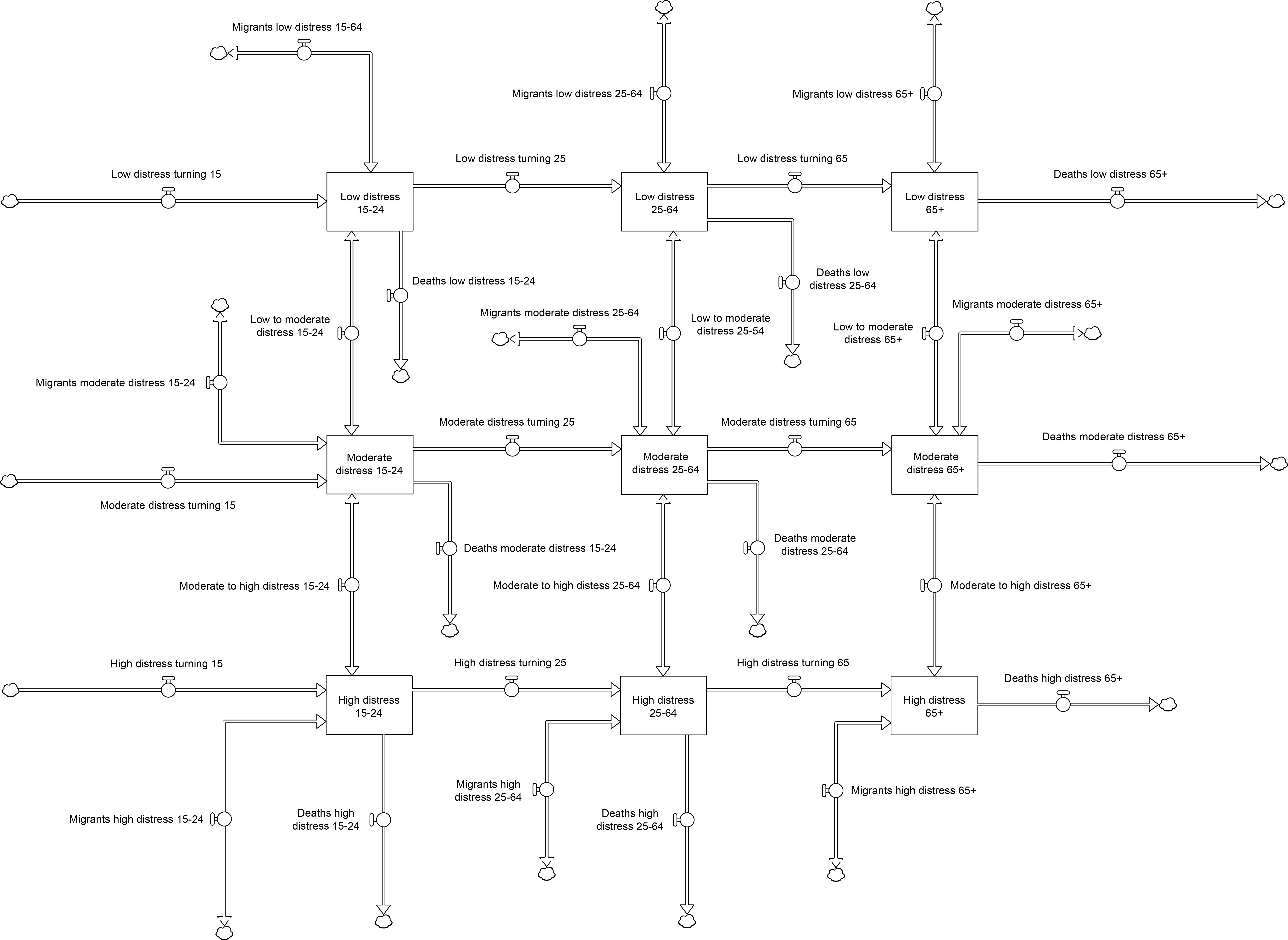


Figure S4. Stock and flow structure of the psychological distress sector.

people in the lower and higher states of distress, respectively, $s$ is the reference (or base) per capita rate at which people transition from the lower state of distress to the higher state of distress per year, $h$ is the product of the effects of adverse childhood experiences (Björkenstam et al., 2015), unemployment (Australian Bureau of Statistics, 2012a), substance use disorders (Marmorstein et al., 2010), intimate partner violence (Ouellet-Morin et al., 2015), and homelessness (Australian Bureau of Statistics, 2012a) on transitions to the higher level of distress, $r$ is the per capita (spontaneous) recovery rate per year, and $T$ is the number of people moving from the higher state of distress to the lower state of distress as a result of effective mental health treatment per year. Note that where $rH+T$ is greater than $hsL$, the flow is negative and there is a net movement of people from the higher level of distress to the lower level of distress.

Numbers of people in each psychological distress category increase (or decrease) through net migration at age-specific rates $p_{ij}I_{i}-q_{ij}e_{i}P_{i}$, where $p_{ij}$ and $q_{ij}$ are the age-specific proportions of people with psychological distress level $j$ in the total state population and the North Coast PHN population, respectively, $I_{i}$ is total age-specific immigration per year, $e_{i}$ is the age-specific per capita emigration rate per year, and $P_{i}$ is the number of people in age group $i$ in the North Coast PHN population. Age-specific per capita mortality rates for people with moderate and high to very high psychological distress are assumed to be 1.16 and 1.37 times those for people with low psychological distress (Russ et al., 2012). Figure S5 presents estimates of the prevalence of moderate and high to very high psychological distress in the North Coast PHN population derived from the system dynamics model and from the NSW Population Health Survey (see http://www.healthstats.nsw.gov.au/).

*1.4. Early life sector*

The early life sector models exposure to adversity in childhood and its effect on the risk of developing mental disorders in adolescence and adulthood. The population of children aged 0−14 years is divided among three stocks, corresponding to states of low, moderate, and high risk of mental illness in later life; transitions to higher levels of risk are assumed to depend on total (cumulative) exposure to adverse experiences (e.g., poverty, physical and sexual abuse, domestic violence), and are irreversible (i.e., transitions only occur from a state of low risk to a state of moderate risk, and from a state of moderate risk to a state of high risk; figure S6). For simplicity (and in the absence of relevant empirical data), we assume that all children are born with low risk of developing a mental disorder, although we acknowledge that levels of risk at birth may vary depending on a range of prenatal and perinatal factors (e.g., Kim et al., 2015). Rates of transition to higher levels of risk are


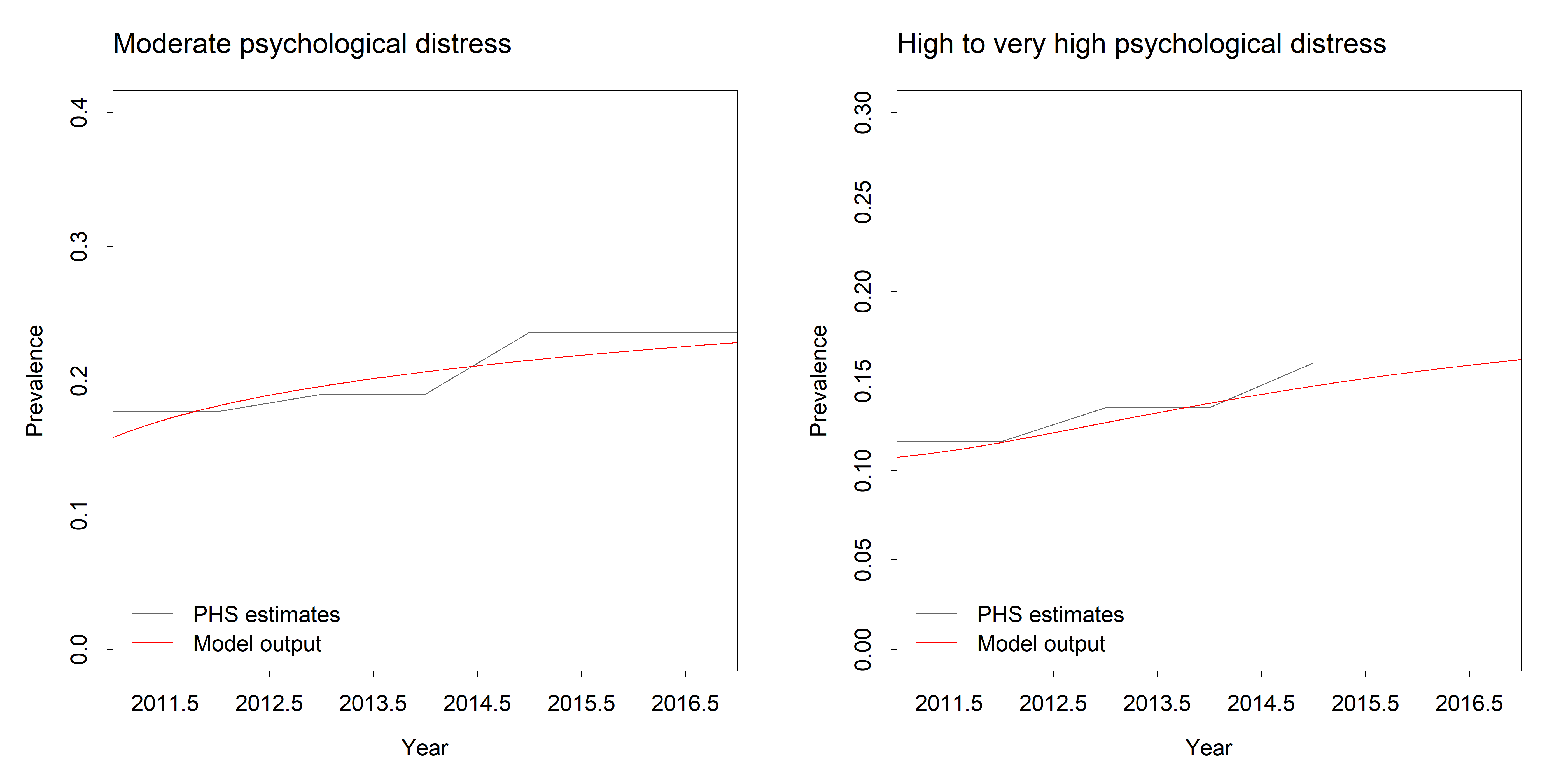


Figure S5. Moderate and high to very high psychological distress prevalence estimates for the total population of the North Coast PHN catchment derived from the model and corresponding NSW Population Health Survey (PHS) estimates.

equal to $hsR$, where $R$ is the number of 0−14-year-olds in the lower state of risk (i.e., low or moderate risk), $s$ is the reference per capita rate at which children transition from the lower state of risk to the higher state of risk per year, and $h$ is the product of the effects of parental psychological distress (Weissman et al., 1997), parental substance abuse (Lynskey et al., 1994), physical and sexual abuse (Spinhoven et al., 2016), and exposure to domestic violence (Kernic et al., 2003) on the risk of developing mental disorders in adolescence and adulthood. The reference (i.e., base) transition rates, $s$, were set so that the proportions of adolescents turning 15 with moderate and high levels of risk aligned with estimates of moderate and high to very high psychological distress prevalence derived from Lawrence et al. (2015) and the NSW School Students Health Behaviours Survey (see figure S7).

As adolescents with moderate and high levels of risk turn 15, they flow into an aging chain comprising stocks of people aged 15−24 years, 25−64 years, and 65 years and above with the same (moderate or high) level of risk. These aging chains provide a means of tracking the numbers of adolescents and adults aged 15 years and above at moderate and high risk of developing mental disorders due to adverse exposures during childhood. A person’s level of risk at the time they reach 15 years of age is assumed to remain unchanged (people are removed from the aging chains only through mortality and emigration) and affects the probability that they will


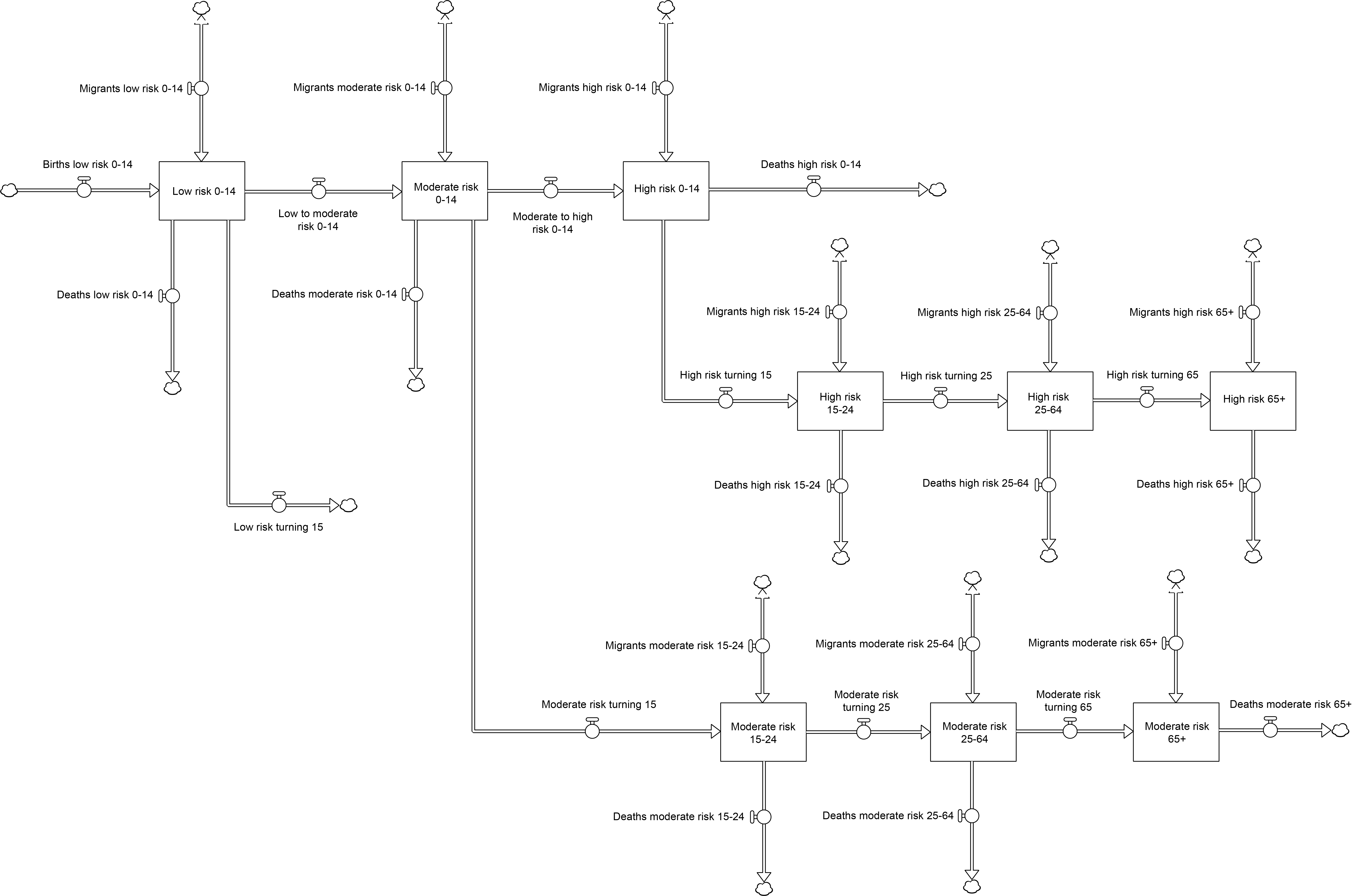


Figure S6. Stock and flow structure of the early life sector.

experience moderate or high to very high psychological distress throughout their life (see section 1.3). Age-specific per capita mortality rates for people with moderate and high levels of risk of mental disorder are assumed to be 1.16 and 1.37 times those for people with a low level of risk (Russ et al., 2012). Net migration adds to (or subtracts from) the numbers of people in each risk state at age-specific rates $p_{ij}I_{i}-q_{ij}e_{i}P_{i}$, where $p_{ij}$ and $q_{ij}$ are the age-specific proportions of people with risk level $j$ in the state population and the North Coast PHN population, respectively, $I_{i}$ is total age-specific immigration per year, $e_{i}$ is the age-specific per capita emigration rate per year, and $P_{i}$ is the number of people in age group $i$ residing in the North Coast PHN catchment.


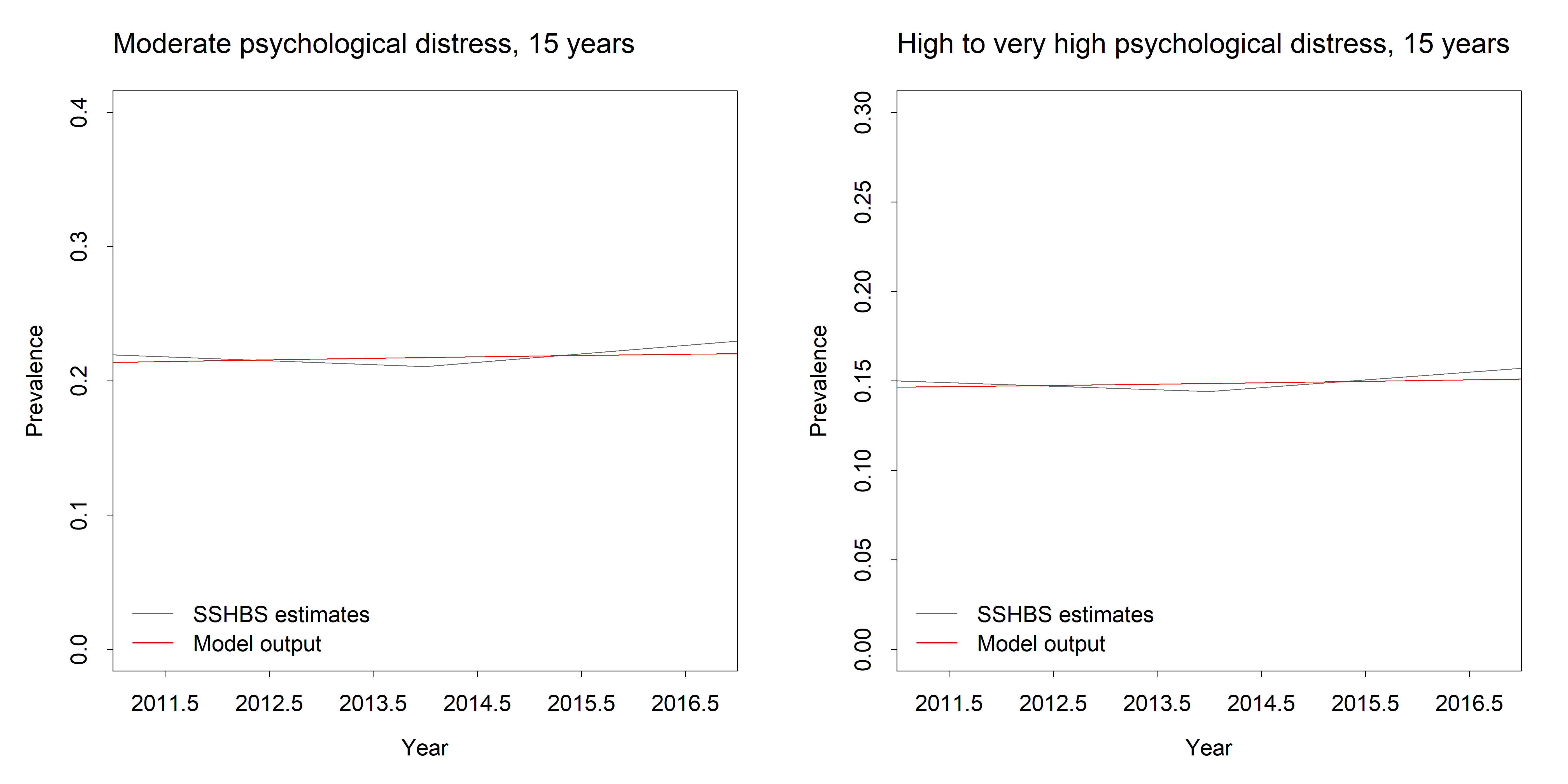


Figure S7. Moderate and high to very high psychological distress prevalence estimates for adolescents aged 15 years derived from the system dynamics model and from the NSW School Students Health Behaviours Survey (SSHBS).

*1.5. Employment sector*

The structure of the employment sector, which captures changes in labour force status in the population aged 15 years and above, is presented in figure S8. The total labour force is represented as six stocks, corresponding to the numbers of employed and unemployed people aged 15−24 years, 25−64 years, and 65 years and above. Adolescents turning 15 are assumed to enter the population of those not in the labour force (NILF), i.e., people who are neither employed nor seeking employment (all 15-years-olds are required to attend school full-time in NSW, and the vast majority will not be in the labour force). People not in the labour force may decide to seek employment, at which point they enter the stocks of unemployed people, while those seeking employment (i.e., the unemployed) may leave the labour force; age-specific net flows from the unemployed population to the population of people not in the labour force are calculated as $fsU-hrN$, where $U$ and $N$ are, respectively, the numbers of unemployed people and people not in the labour force, $s$ is the reference (base) per capita rate that unemployed people leave the labour force per year, $f$ is the effect of the unemployment rate on labour force participation (assumed to be greater than 1, so that increased unemployment reduces participation; see Mitchell et al., 2019), $r$ is the base per capita rate that people enter the labour force per year, and $h$ is the product of the effects of psychological distress (Frijters et al., 2014) and substance abuse disorders (Zabkiewicz and Schmidt, 2007) on the labour force entry rate.


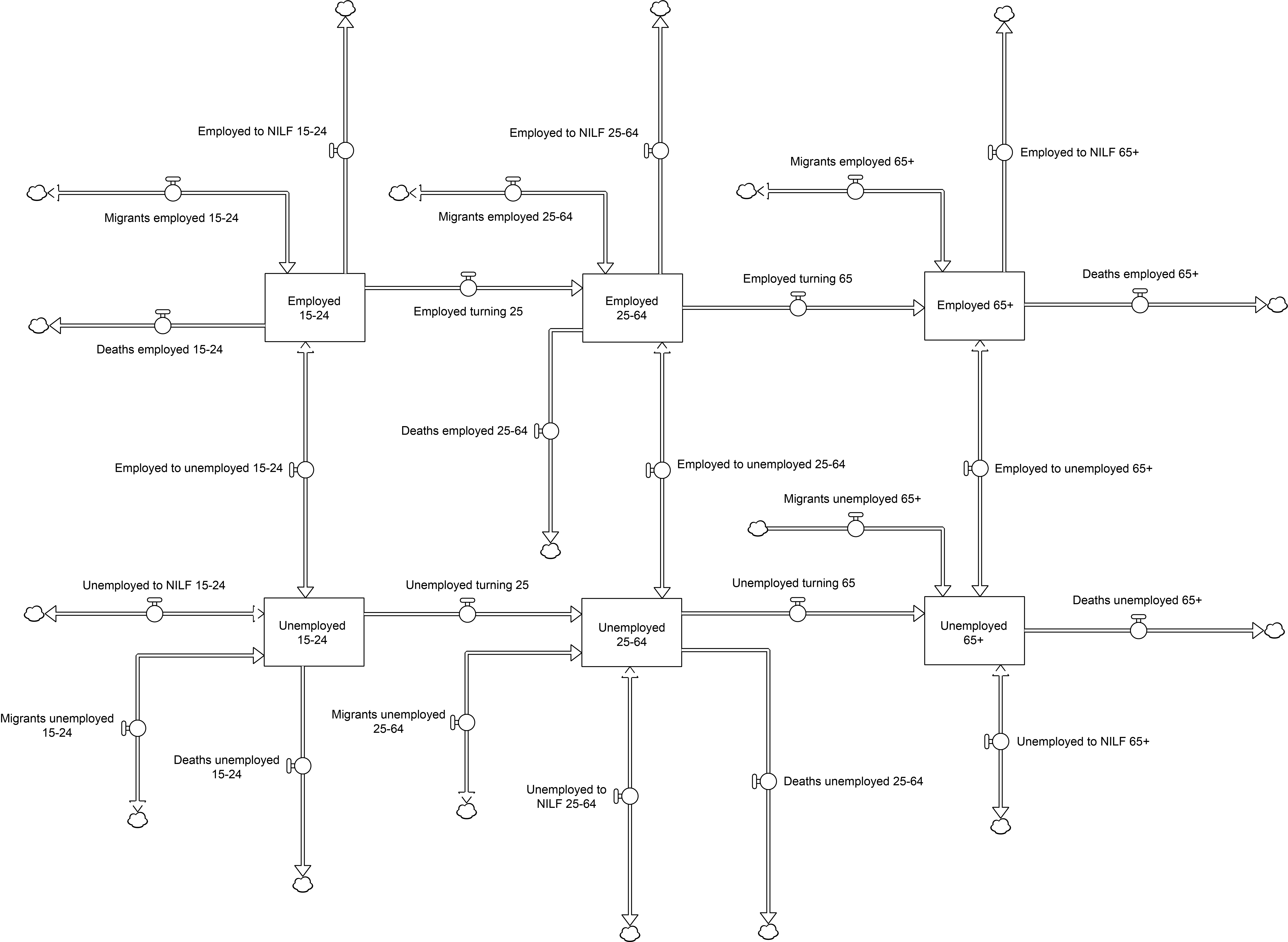


Figure S8. Stock and flow structure of the employment sector.

Employed people become unemployed at age-specific rates equal to $vE$, where $E$ is the employed population and $v$ is the per capita rate of job loss per year, while unemployed people secure employment at age-specific rates $fwU$, where $U$ is the unemployed population, $w$ is the base per capita rate of employment initiation per year, and $f$ is the product of the effects of psychological distress (Frijters et al., 2014) and substance abuse disorders (Zabkiewicz and Schmidt, 2007) on the employment initiation rate (assumed to be the same as the effects on labour force participation; see previous paragraph); the net yearly flow from employment to unemployment is therefore $vE-fwU$. People in employment leave the labour force (e.g., due to retirement or parenting responsibilities) at age-specific rates $\gamma_{i}zE_{i}$, where $E_{i}$ is the number of employed people in age group $i$, $z$ is the per capita rate that employed 15−24-year-olds leave the labour force per year, and $\gamma_{i}$ is the effect of being in age group $i$ on the labour force exit rate (equal to 1 for 15−24-year-olds). Net migration increases (or depletes) the employed population at rates equal to $p_{i}I_{i}-q_{i}e_{i}P_{i}$, where $p_{i}$ and $q_{i}$ are the age-specific employment rates for the total state population and the North Coast PHN population, respectively, $I_{i}$ is total age-specific immigration per year, $e_{i}$ is the age-specific per capita emigration rate per year, and $P_{i}$ is the number of people in age group $i$ residing in the North Coast PHN catchment (the effect of net migration on the unemployed population is modelled in the same way). Per capita mortality rates for people seeking employment are assumed to be 1.22 times those for people who are employed or not in the labour force (Sorlie and Rogot, 1990). Figure S9 presents participation and unemployment rate estimates derived from the system dynamics model and from official labour force data published by the Australian Bureau of Statistics (Australian Bureau of Statistics, 2019a).

*1.6. Substance abuse sector*

The substance abuse sector captures the incidence of substance abuse disorders and the flow of patients through alcohol and other drug (AOD) treatment services (see figure S10). People aged 15−24 years and 25 years or more with chronic substance abuse disorders are divided among stocks corresponding to those not engaged with AOD treatment services, patients waiting for treatment services, and patients receiving treatment. New substance abuse disorder cases and people relapsing after treatment are added to the stocks of those not engaged with treatment services at age-specific rates $hsG+\left( 1-r \right)T$, where $G$ is the number of people without a substance abuse disorder, $s$ is the base per capita rate at which people develop substance use disorders per year, $h$ is the product of the effects of psychological distress (Marmorstein et al., 2010), adverse experiences in childhood (Crum et al., 2008), and homelessness (Johnson et al., 1997) on substance abuse disorder incidence, $T$ is the number of people completing treatment per year, and $r$ is the proportion of people recovering after treatment (24.1%; Miller et al., 2001). People with substance use disorders not engaged with treatment services flow into the stocks of those waiting for services at age-specific per capita rates that remain constant over the simulation period. Rates of treatment initiation depend on services capacity (i.e., the number of closed treatment episodes that can be provided per year), which is equal to $C_{0}+\beta t$, where $C_{0}$ is services capacity at the start of the simulation period (i.e., 1 January 2011), $\beta$ is the increase in capacity per year (estimated from services usage data for 2013−14 to 2016−17; Australian Institute of Health and Welfare, 2018a), and $t$ is time in years since the simulation start date. Patients waiting for treatment are assumed to disengage from services at a constant per capita rate estimated from data in Stevens et al. (2008).


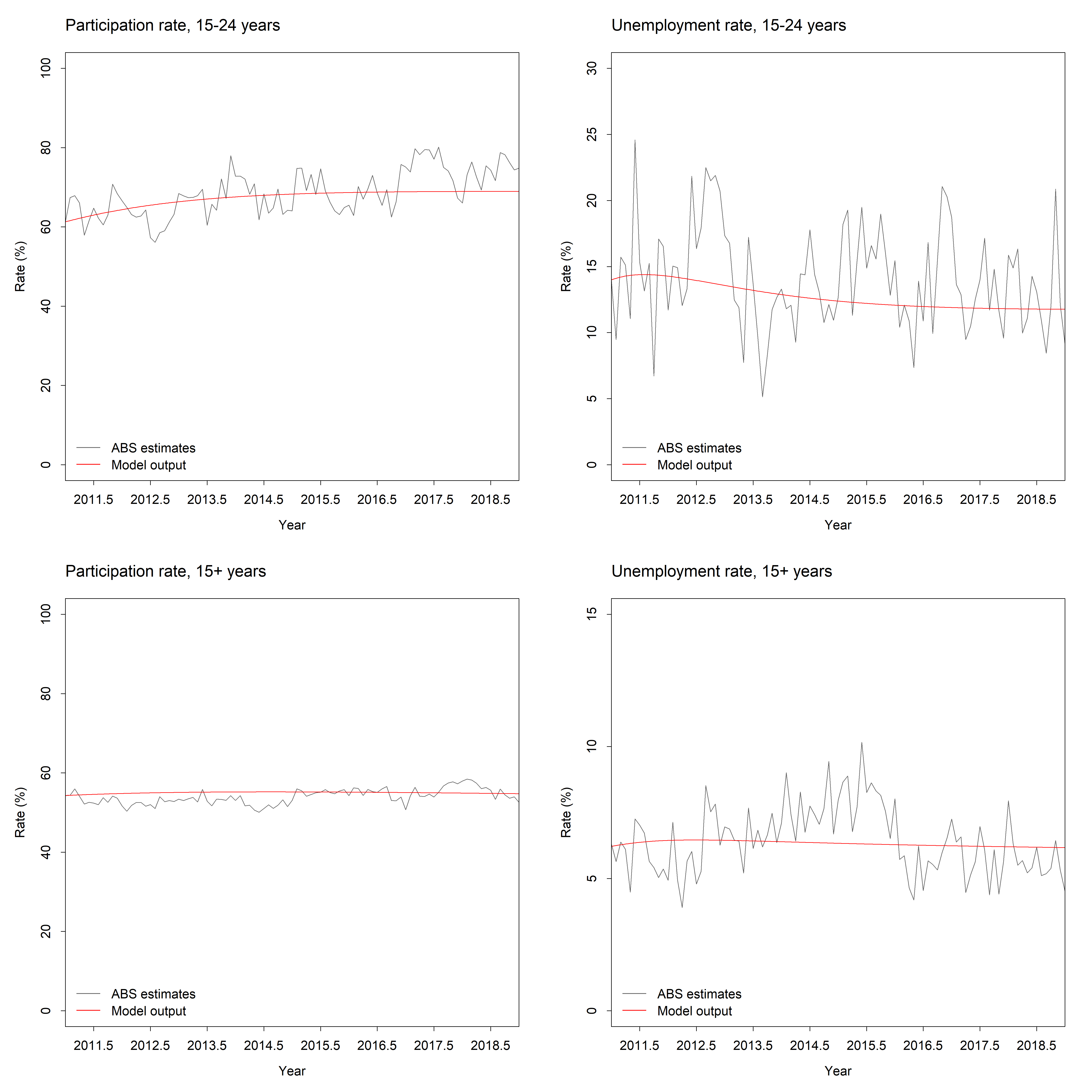


Figure S9. Participation and unemployment rate estimates derived from the system dynamics model and from Australian Bureau of Statistics (ABS) labour force data.


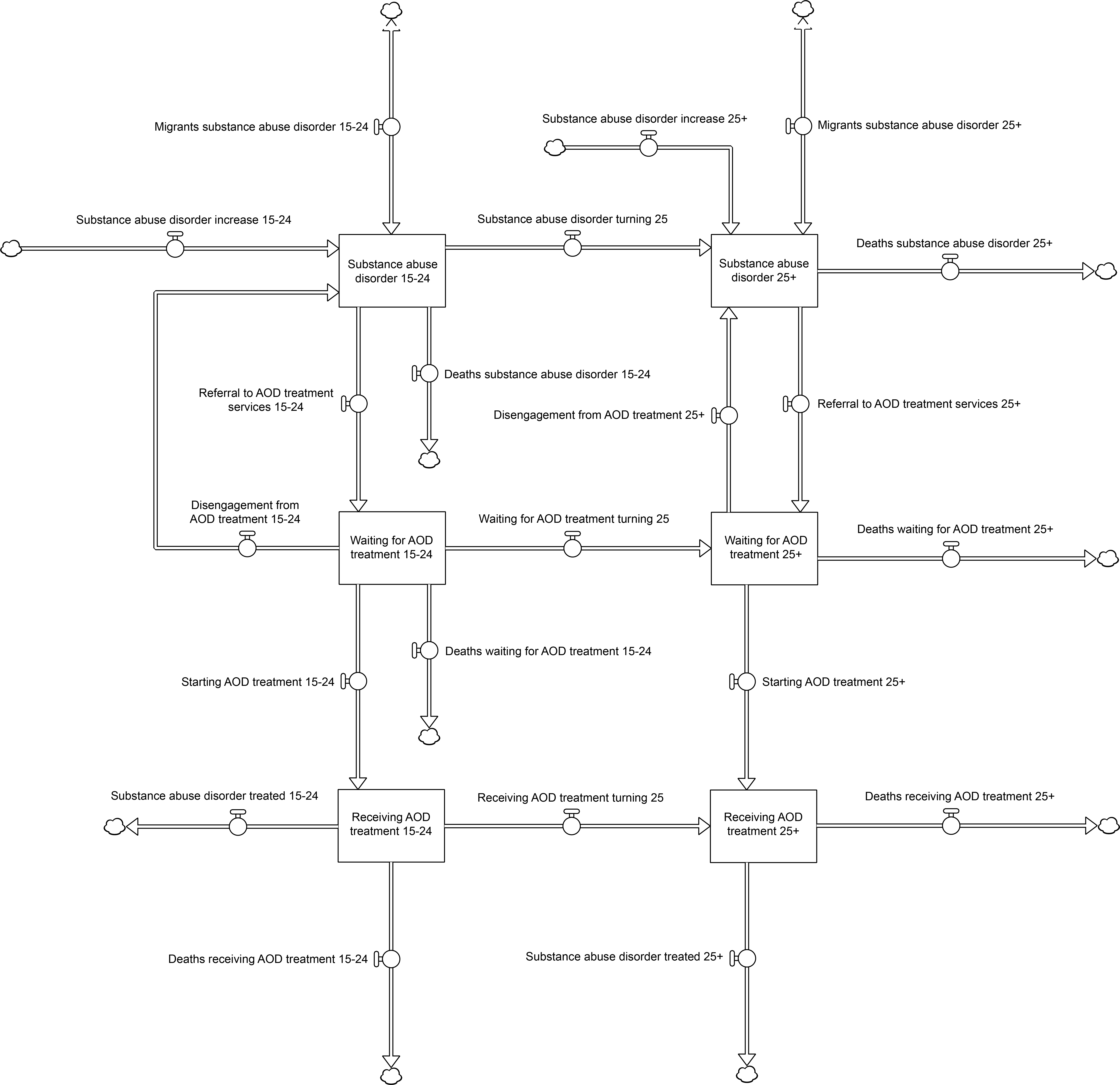


Figure S10. Stock and flow structure of the substance abuse disorder sector.

Rates of treatment completion are equal to $M/d$, where $M$ is the number of patients receiving treatment at any particular time point and $d$ is the mean duration of care (19 days, or 0.052 years; Australian Institute of Health and Welfare, 2018a). A constant proportion of patients completing treatment recover (24.1%; Miller et al., 2001), returning to the general population (i.e., people without a substance abuse disorder), while the remaining patients flow directly into the stocks of those with substance use disorders who are not currently engaged with AOD treatment services (see above). Net migration adds to (or subtracts from) the stocks of people with substance use disorders who are not engaged with services at age-specific rates $p_{i}I_{i}-e_{i}S_{i}$, where $p_{i}$ is the age-specific prevalence of substance abuse disorders in the state population, $I_{i}$ is total age-specific immigration per year, $e_{i}$ is the age-specific per capita emigration rate per year, and $S_{i}$ is the number of North Coast PHN residents in age group $i$ who have a substance abuse disorder and are not engaged with treatment services. Age-specific per capita mortality rates for people with substance abuse disorders are assumed to be 1.95 times those for the general population (Roerecke and Rehm, 2013). Figure S11 presents model-based estimates of the prevalence of substance abuse disorders and treatment services usage rates together with estimates derived from the NSW Population Health Survey (see http://www.healthstats.nsw.gov.au/), the National Health Survey (Australian Bureau of Statistics, 2015), and services usage data published by the Australian Institute of Health and Welfare (2018a).

*1.7. Domestic violence and homelessness sectors*

The structure of the domestic violence sector is shown in figure S12. Age-specific base rates of intimate partner violence are modelled as stocks that increase (65+ years) or decrease (15−24 years and 25−64 years) at a constant rate per year. The incidence of intimate partner violence in age group $i$ is equal to $f_{i}v_{i}$, where $v_{i}$ is the base number of incidents per year and $f_{i}$ is the product of the effects of partner substance abuse and partner unemployment on the domestic violence rate (Kyriacou et al., 1999). Rates of increase (or decrease) in the base rates, $v_{i}$, were set so that the incidence of intimate partner violence in each age group aligned with estimates derived from Australian Bureau of Statistics data on domestic violence-related offences (Australian Bureau of Statistics, 2019b; see figure S13).

Figure S14 presents the structure of the homelessness sector, which models age-specific transitions into and out of homelessness. The homeless population of the North Coast PHN catchment is represented as four stocks, corresponding to people aged 0−14 years, 15−24 years, 25−64 years, and 65 years and above satisfying the statistical definition of homelessness developed by the Australian Bureau of Statistics (2012b). People flow into these stocks at age-specific rates equal to $r_{i}h_{i}N_{i}+qs_{i}P_{i}$, where $P_{i}$ and $N_{i}$ are, respectively, the total number of people in age group $i$ and the number of people in age group $i$ with secure housing, $h_{i}$ is the reference (i.e., base) per capita rate at which people in age group $i$ with secure housing become homeless per year (for reasons other than domestic violence), $r_{i}$ is the product of the age-specific effects of childhood adversity (Herman et al., 1997) and substance use disorders (Shelton et al., 2009) on the risk of homelessness, $s_{i}$ is the age-specific domestic violence rate, and $q$ is the probability of becoming homeless after a domestic violence incident (0.023; Australian Bureau of Statistics, 2017). Homelessness declines as people secure housing at age-specific rates


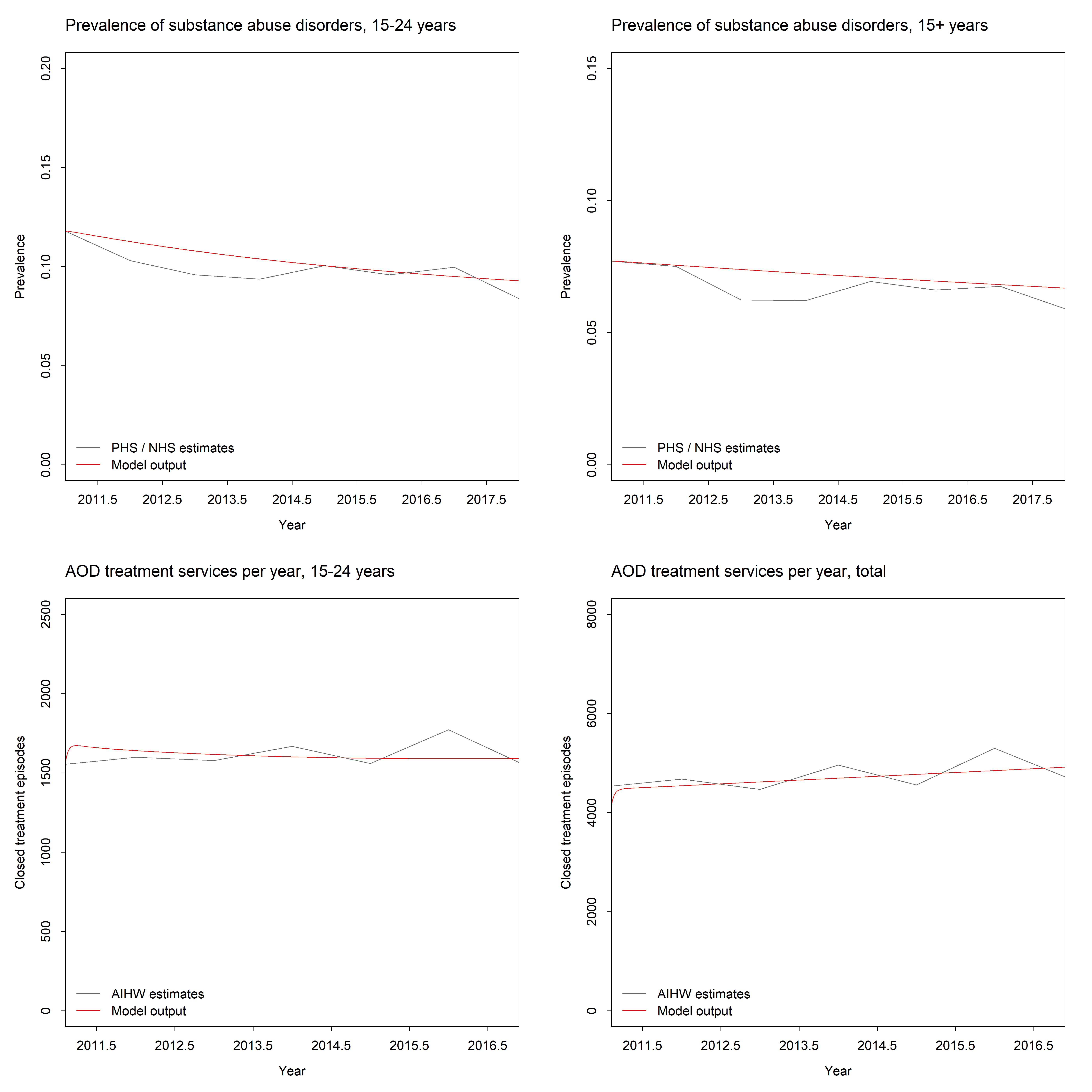


Figure S11. Estimates of the prevalence of substance abuse disorders and treatment services usage rates derived from the system dynamics model and from the NSW Population Health Survey (PHS), the National Health Survey (NHS), and services usage data published by the Australian Institute of Health and Welfare (AIHW).

${H_{i}}/d$, where $H_{i}$ is the homeless population for age group $i$ and $d$ is the mean duration of homelessness (20.4 weeks, or 0.392 years; Australian Bureau of Statistics, 2016). Per capita mortality rates for homeless people are assumed to be 1.60 times those for people with secure housing (Morrison, 2009). Estimates of the homeless population derived from the model are presented alongside Australian Bureau of Statistics (2018) estimates in figure S13.


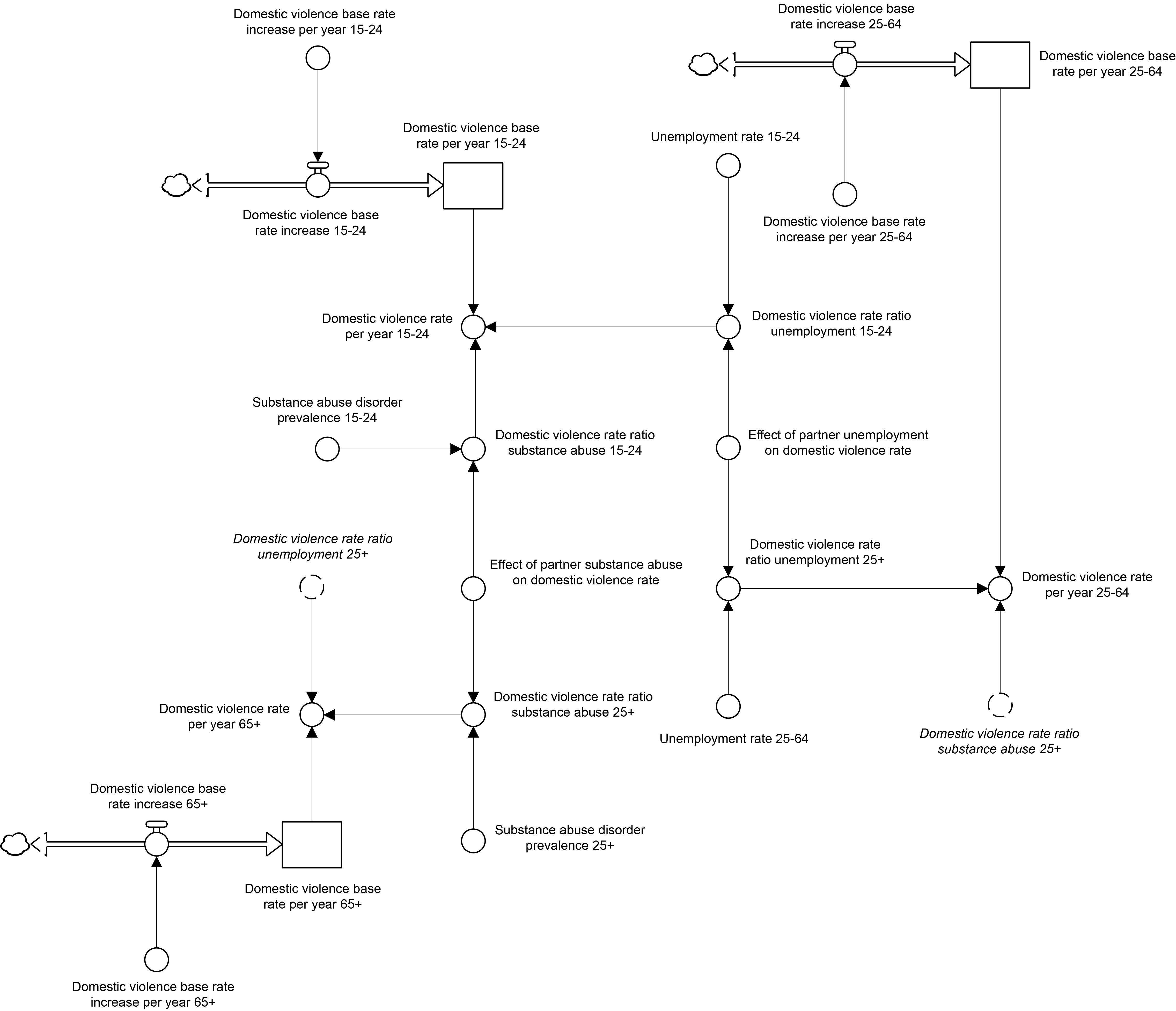


Figure S12. Structure of the domestic violence sector.


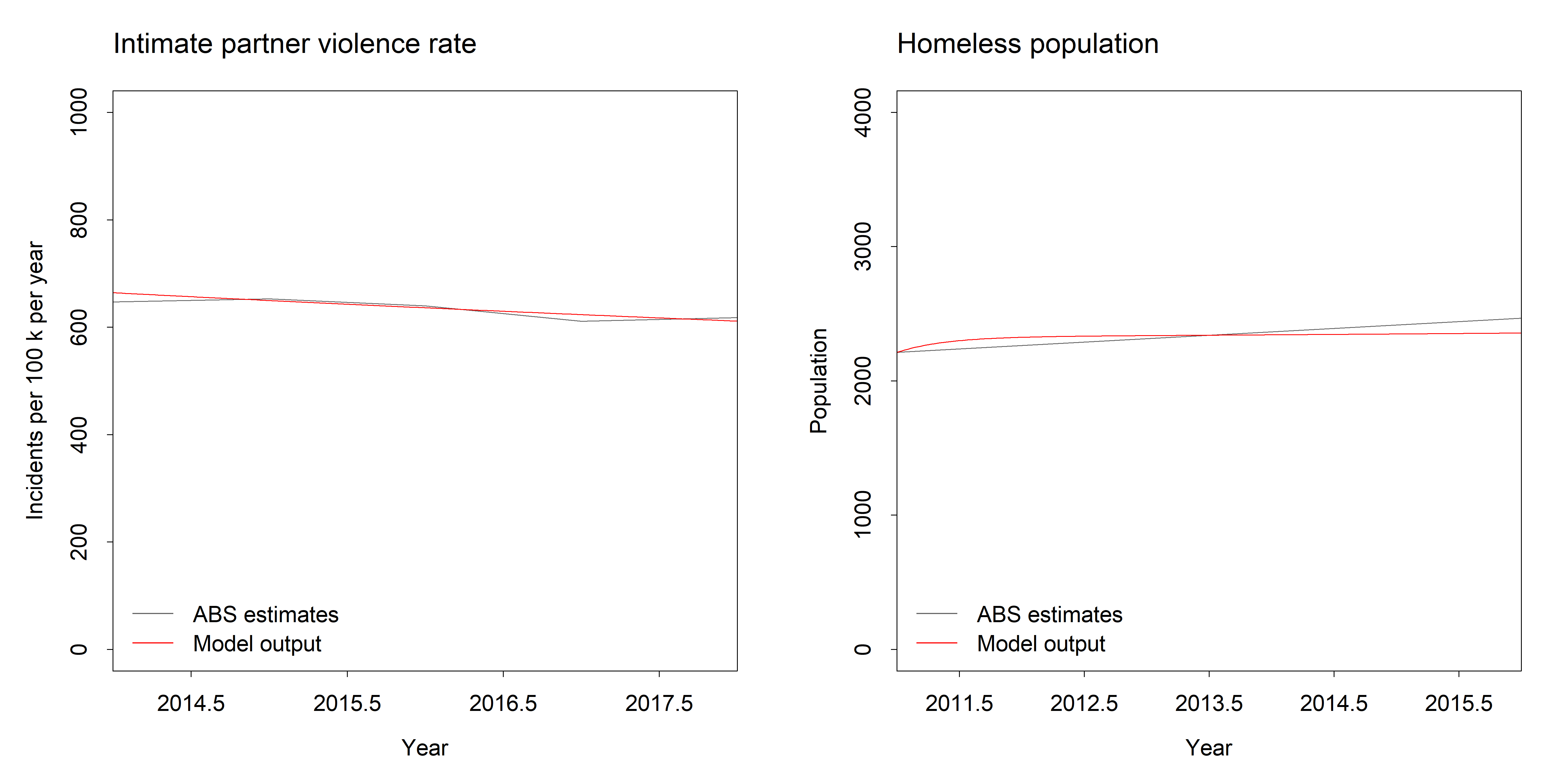


Figure S13. Estimates of the incidence of intimate partner violence and the homeless population derived from the system dynamics model and from Australian Bureau of Statistics (ABS) data.


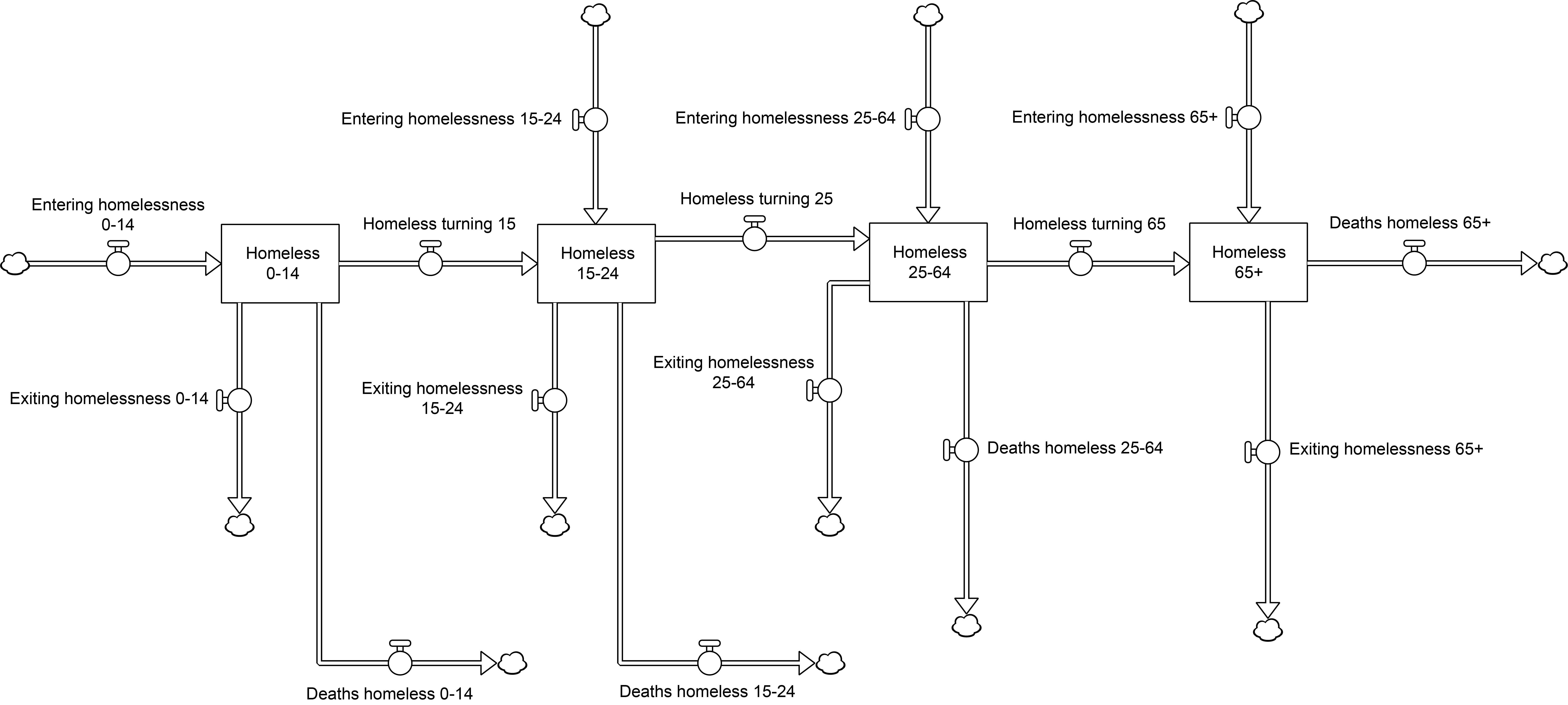


Figure S14. Stock and flow structure of the homelessness sector.

*1.8. Mental health services sector*

Figure S15 presents a high-level map of the mental health services sector, which models the flow of distressed patients through the mental health care system. People with moderate or high to very high psychological distress engage with mental health services in one of two ways; they may perceive a need for mental health care and seek help (e.g., from a general practitioner or online services), or they may present to an emergency department (e.g., for self-harm) without having previously perceived a need for treatment. After engaging with mental health services, patients may recover following treatment, returning to the general population with a low or moderate level of psychological distress (depending on their prior level of distress), be treated but not recover, or disengage due to excessive waiting times (a result of insufficient services capacity) or because they are dissatisfied with the care they receive. Patients who are treated but do not recover return to perceiving a need for services and will eventually seek help again if they do not recover spontaneously; thus, psychologically distressed people entering the mental health services system continue receiving treatment (modelled as individual service contacts; see below) until they recover, disengage, or die (mortality is captured in the model, but is not shown in figure S15). The principal components of the mental health services sector are described in detail below.

*Help seeking* — People experiencing moderate or high to very high psychological distress who are not currently considering engaging with mental health services perceive a need for care at rates equal to $p_{i}D_{i}$, where $D_{i}$ is the


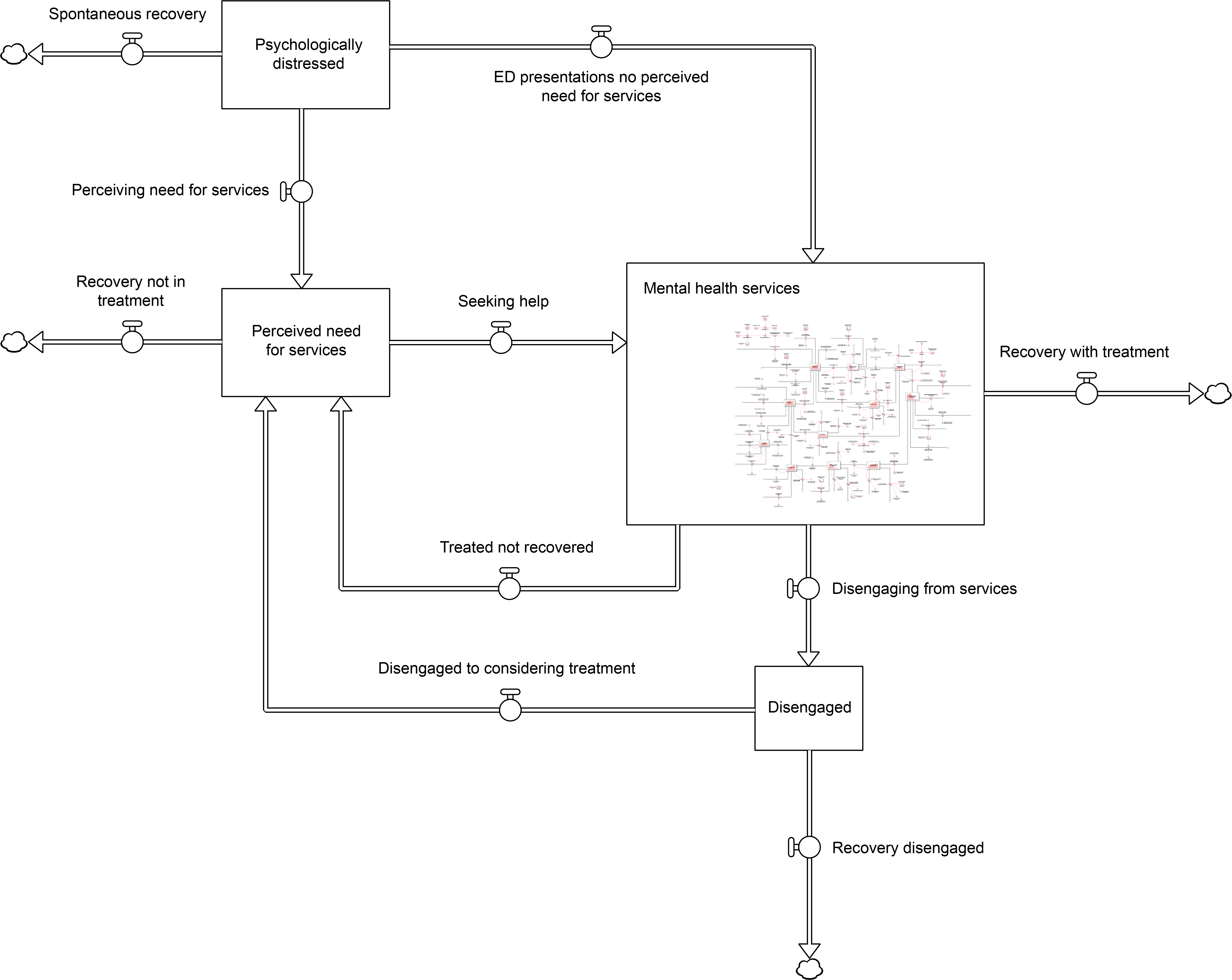


Figure S15. High-level map of the mental health services sector.

number of people with distress level $i$ not currently considering treatment and $p_{i}$ is the per capita rate that people with distress level $i$ perceive a need for care per year. The per capita rates $p_{i}$ are assumed to increase at a constant rate per year due to increasing public awareness of high-prevalence mental disorders and available treatment options. After perceiving a need for treatment, people engage with mental health services at per capita rates that depend on their age and state of psychological distress (Australian Bureau of Statistics, 2012a). Recently treated patients who have not recovered or disengaged from services return to perceiving a need for care and may attend subsequent (planned or unplanned) appointments with a GP or community-based psychiatric services (i.e., psychiatrists and allied health services, hospital outpatient services), be admitted to a general hospital, commence online treatment, or present to an emergency department (e.g., for suicidal ideation); the stocks of people perceiving a need for services (figure S16) therefore contain a mix of prospective patients and patients already engaged with the mental health care system. These prospective and current patients may age, recover spontaneously, or transition from a state of moderate distress to a state of high or very high distress, and are assumed to experience the same per capita mortality rates as similar-aged people with the same level of psychological distress who are not considering treatment.

*GP and online services* — People with a perceived need for mental health services seek help from a GP at age-specific rates $\theta_{j}s_{i}P_{ij}$, where $P_{ij}$ is the number of people in age group $i$ with distress level $j$ perceiving a need for care (those in the arrayed stock labelled ‘Perceived need for services’; figure S16), $s_{i}$ is the age-specific per capita rate at which people with moderate psychological distress and a perceived need for care seek help from a GP, and the rate ratio $\theta_{j}$ is equal to 1 for people with moderate distress and less than 1 for people with high to very high distress (i.e., people with high to very high psychological distress are assumed to seek help at a lower rate than those with moderate distress, given a perceived need for care; see Australian Bureau of Statistics, 2012a). The per capita rates $s_{i}$ are assumed to increase at a constant rate per year, increasing help seeking rates among people who perceive a need for treatment. Prior to receiving care, patients seeking help from a GP or referred to GP services after completing hospital inpatient care wait for a varying period of time that depends on services capacity and the total number of patients waiting for care. GP services capacity, i.e., the number of mental health-related GP consultations that can be provided per year, is assumed to increase at a constant rate per year, estimated from Medicare Benefits Schedule (MBS) claims data for the period 2012−2017 (figure S17).

Mental health-related GP consultations are represented as a stock (arrayed by age group and distress level) with outflows corresponding to treatment completion, referral to other services (including psychiatrist and allied health services, community mental health care services, and online services), and disengagement resulting from dissatisfaction with the care provided. Age-specific recovery rates among patients completing treatment are equal to $v\gamma_{j}rT_{ij}$, where $T_{ij}$ is the number of patients in age group $i$ with distress level $j$ completing treatment per year, $v$ is the proportion of patients completing treatment who receive psychological therapy, $r$ is the proportion of patients with moderate psychological distress who recover after receiving psychological therapy, and the recovery rate ratio $\gamma_{j}$ is equal to 1 for moderately distressed patients and less than 1 for patients experiencing high to very high psychological distress (i.e., psychological therapy provided by a GP is assumed to be less effective for highly distressed patients than for moderately distressed patients; see Cuijpers et al., 2009). Patients completing treatment who do not recover return to the stocks of people who perceive a need for care.


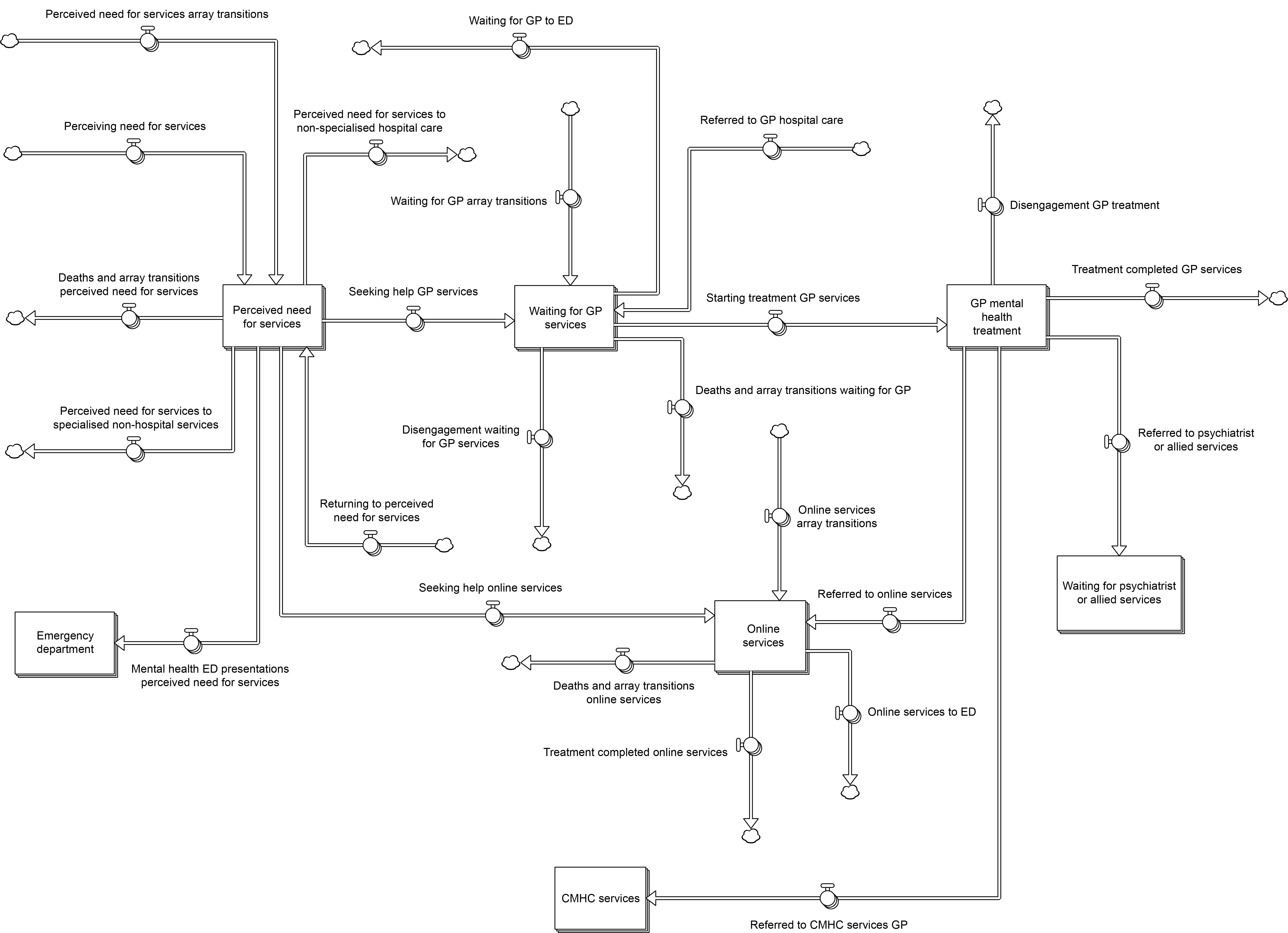


Figure S16. Stock and flow structure of the help-seeking, general practitioner (GP) services, and online services components of the mental health services sector.


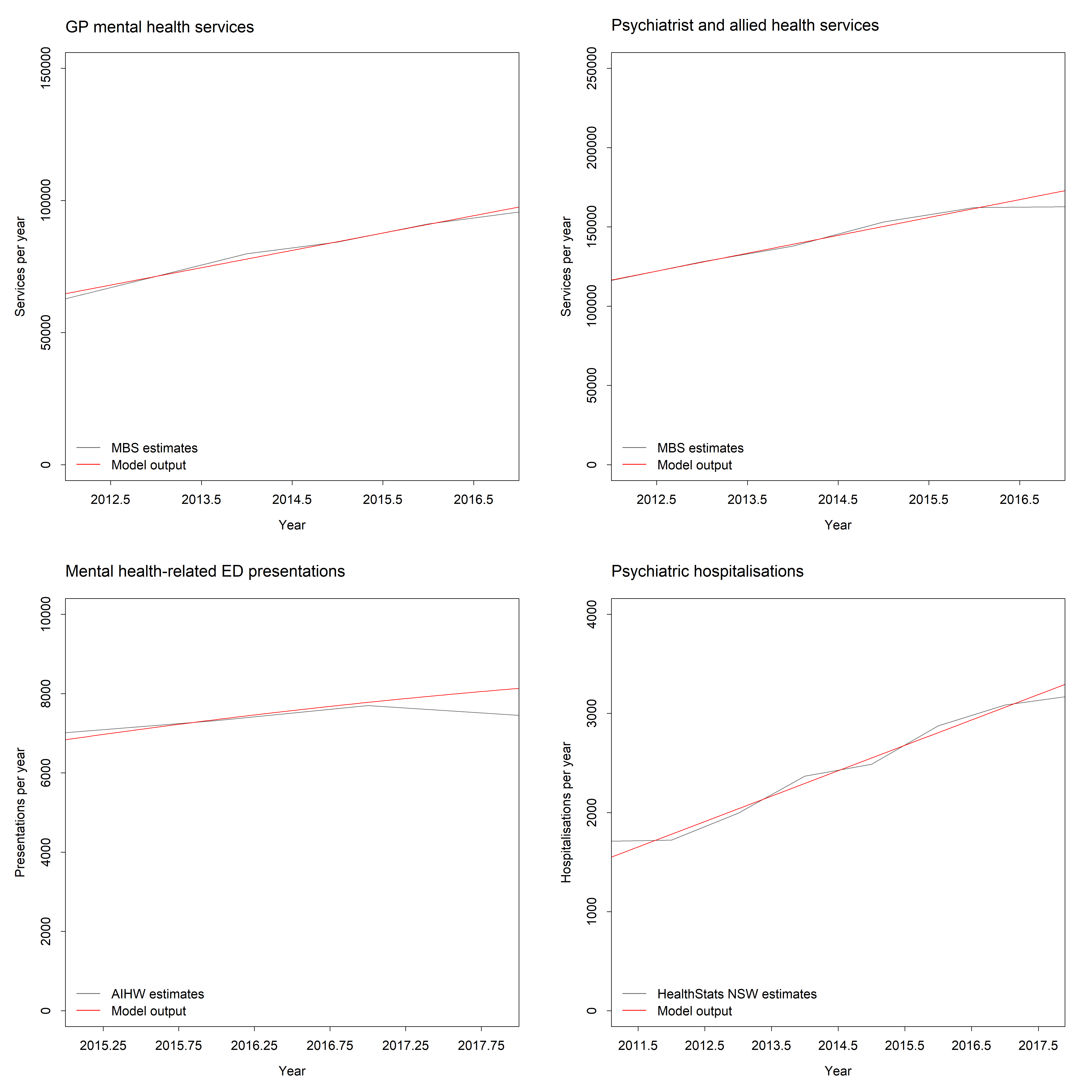


Figure S17. Mental health services usage rates derived from the system dynamics model and from Medicare Benefits Schedule (MBS) data, data published by the Australian Institute of Health and Welfare (AIHW), and data available from HealthStats NSW (http://www.healthstats.nsw.gov.au/).

Age-specific treatment commencement rates for online (self-help) services are equal to $h_{j}P_{ij}+uG_{ij}$, where $P_{ij}$ is the number of people in age group $i$ with psychological distress level $j$ who perceive a need for care (i.e., those in the arrayed stock labelled ‘Perceived need for services’ in figure S16), $h_{j}$ is the per capita rate that people with distress level $j$ and a perceived need for care access online services per year, $G_{ij}$ is the GP services provision rate (i.e., the number of patients in age group $i$ with psychological distress level $j$ attending a GP consultation per year), and $u$ is the fraction of patients visiting a GP for a mental health issue referred to online services. Prior to completing treatment, people accessing online services may recover spontaneously, present to an emergency department, or die (see figure S16); treatment completion rates are equal to ${O_{ij}}/d$, where $O_{ij}$ is the number of people in age group $i$ with distress level $j$ engaged in online treatment and $d$ is the mean duration of online treatment programs (assumed to be 6 weeks, or 0.115 years; Christensen et al., 2004). People completing online treatment programs recover at rates equal to $\eta_{j}z{O_{ij}}/d$, where $z$ is the fraction of people with moderate psychological distress completing treatment who recover and the rate ratio $\eta_{j}$ is equal to 1 for moderately distressed people and significantly less than 1 for people with high to very high distress (i.e., online services are assumed to be more effective for people with moderate psychological distress than for people with high to very high distress).

*Psychiatrist and allied health services* — Figure S18 presents the structure of the psychiatrist and allied health services component of the mental health services sector. Prior to receiving treatment, patients referred to a psychiatrist or allied health services by a GP or after completing hospital inpatient care wait for a period of time that depends on services capacity and the total number of patients waiting for care. The arrayed stock of people waiting for treatment also contains patients currently engaged with specialised services who have planned (follow-up) appointments (these patients and patients referred to services after receiving hospital care enter via the flow labelled ‘Additional psychiatrist or allied health services’; see figure S18). Services capacity, i.e., the number of psychiatrist and allied health services that can be provided per year, increases at a constant rate per year, estimated from MBS claims data for the period 2012−2017 (see figure S17). Patients receiving treatment are referred to psychiatric hospital services, disengage from the mental health services system due to dissatisfaction with the care received, recover, or return to perceiving a need for care (these patients flow back into the arrayed stock labelled ‘Perceived need for services’; see figure S16). Recovery rates are equal to $rT_{ij}$, where $T_{ij}$ is the number of patients in age group $i$ with psychological distress level $j$ who complete treatment per year and $r$ is the fraction of patients recovering after receiving specialised psychiatric care. Psychiatrist and allied health services usage rates derived from the model and from MBS claims data are presented in figure S17.

*Hospital services* — Mental health-related hospital services captured in the model include psychiatric and non-specialised inpatient care, outpatient care delivered by community mental health care (CMHC) teams, and emergency department attendances (see figure S19). Emergency department (ED) presentation rates are equal to $\theta_{j}e_{i}C_{ij}+\theta_{j}f_{i}N_{ij}$, where $C_{ij}$ is the number of people in age group $i$ with psychological distress level $j$


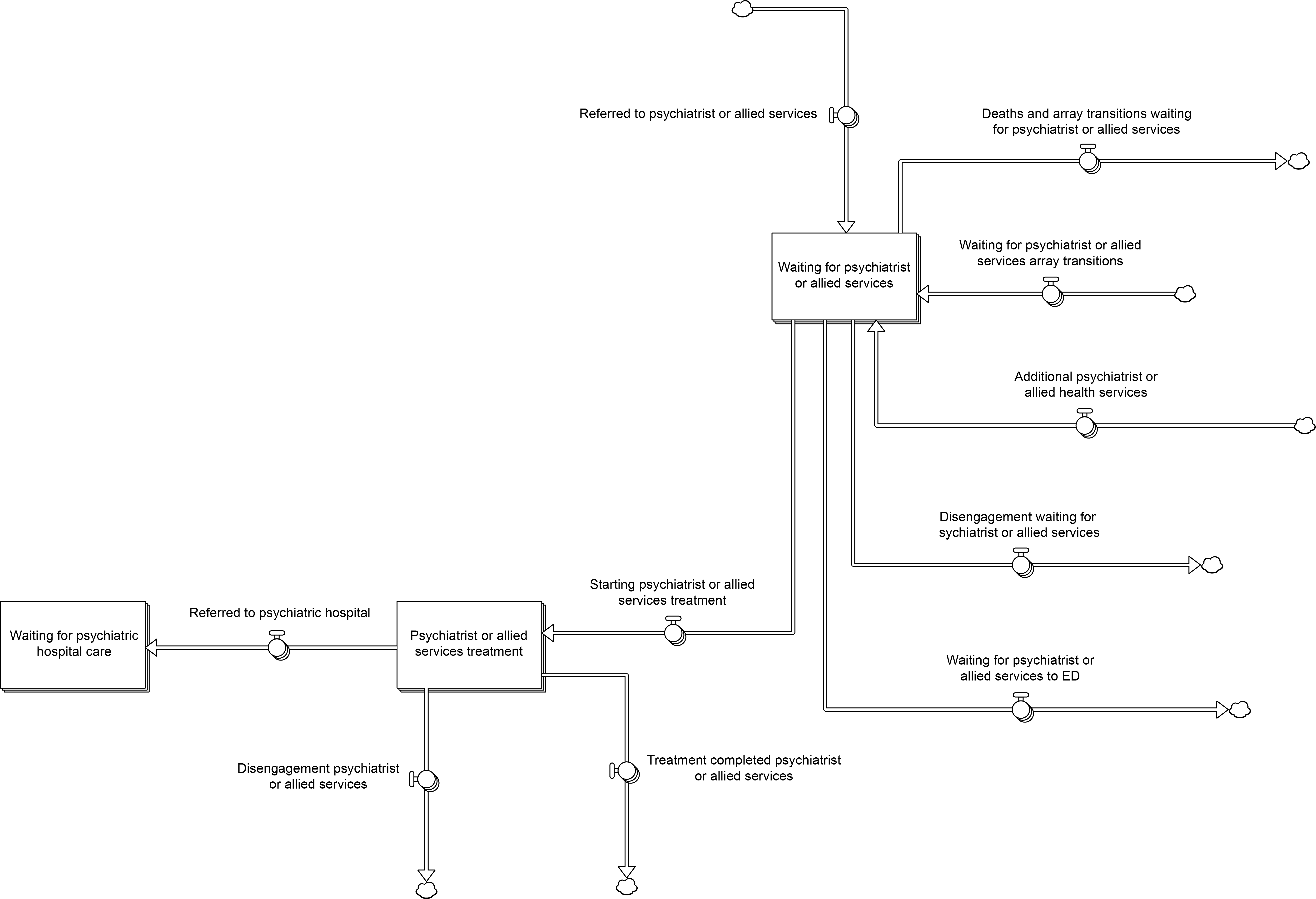


Figure S18. Stock and flow structure of the psychiatrist and allied health services component of the mental health services sector.


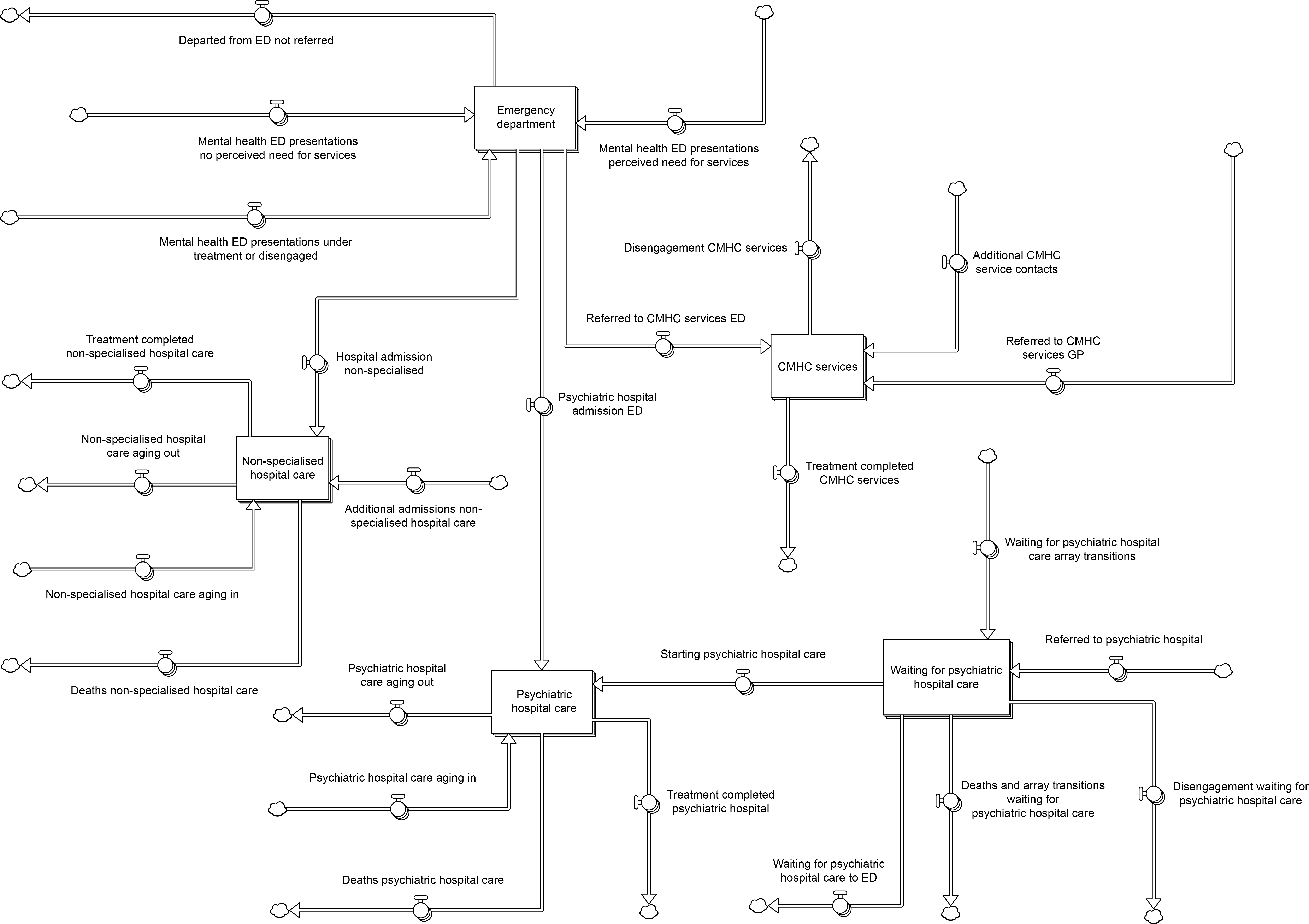


Figure S19. Stock and flow structure of the hospital services component of the mental health services sector.

perceiving a need for care (including all patients waiting for care or engaged in online treatment and those who have disengaged from services; figures S16, S18−S20), $N_{ij}$ is the number of people in age group $i$ with distress level $j$ who do not perceive a need for services, $e_{i}$ and $f_{i}$ are age-specific per capita ED presentation rates for moderately distressed people perceiving a need for care and with no perceived need for care, respectively, and the rate ratio $\theta_{j}$ is equal to 1 for people with moderate psychological distress and more than 1 for people experiencing high to very high distress (i.e., people in a state of high or very high distress are assumed to present to an ED at higher rates than those with a moderate level of distress). People presenting to an ED may be admitted to a psychiatric or general hospital ward, referred to CMHC services, or discharged to the community. A fraction of patients perceiving a need for care who are discharged to the community disengage from services due to dissatisfaction with the care provided.

Psychiatric and general hospital admission rates are constrained by hospital capacity (i.e., the total numbers of patients that can be admitted annually), which is assumed to increase linearly over the simulation period; capacity increase rates per year were estimated from hospital separations data available from HealthStats NSW (see http://www.healthstats.nsw.gov.au/) and data published by the Australian Institute of Health and Welfare (2018b). Psychiatrists refer patients to psychiatric hospital care at rates equal to $\gamma_{j}qM_{ij}$, where $M_{ij}$ is the number of patients in age group $i$ with distress level $j$ receiving psychiatrist and allied health services per year, $q$ is the fraction of moderately distressed patients receiving psychiatrist and allied health services referred to psychiatric hospital care, and the rate ratio $\gamma_{j}$ is equal to 1 for patients with moderate distress and substantially more than 1 for patients with high to very high distress. Prior to being admitted, patients referred to a psychiatric hospital wait for a period of time that depends on hospital capacity, the total number of referred patients waiting for care, and the rate at which patients are being admitted via emergency departments (since available capacity declines as the ED-related admission rate increases). People with a perceived need for services are admitted for general (non-specialised) hospital care without presenting to an ED at per capita rates that depend on age and distress level and remain constant over the simulation period (the flow labelled ‘Additional admissions non-specialised hospital care’ in figure S19). A fraction of patients discharged from psychiatric or general hospital care disengage from services due to dissatisfaction with the care provided; patients who do not disengage from treatment are referred to a GP, psychiatrist and allied health services, or CMHC services for follow-up care.

Age-specific community mental health care (CMHC, or hospital outpatient) services provision rates are equal to $\eta_{j}h_{i}G_{ij}+pE_{ij}+S_{ij}+A_{ij}+k_{j}u_{i}P_{ij}$, where $G_{ij}$ is the number of patients in age group $i$ with distress level $j$ visiting a GP per year, $h_{i}$ is the proportion of moderately distressed patients in age group $i$ visiting a GP who are referred to CMHC services, $E_{ij}$ is the number of people presenting to an ED per year not admitted to hospital, $p$ is the proportion of patients discharged from an ED referred to CMHC services, $S_{ij}$ and $A_{ij}$ are, respectively, the numbers of patients discharged from psychiatric and general hospital inpatient care per year referred to CMHC services, $P_{ij}$ is the number of people with a perceived need for care (those in the arrayed stock labelled ‘Perceived need for services’; figure S16), $u_{i}$ is the per capita CMHC services contact rate among people with a perceived need for care (e.g., for follow-up appointments), and the rate ratios $\eta_{j}$ and $k_{j}$ are equal to 1 for patients with moderate psychological distress and more than 1 for patients with high to very high psychological distress. Patients receiving CMHC services recover, return to the stocks of people perceiving a need for services, or disengage from the mental health care system due to dissatisfaction with the care provided (figure S19). The per-service recovery rate for patients who do not disengage from treatment is calculated as ${rK}/T$, where $K$ is the total number of CMHC services that can be provided per year while maintaining the base (or reference) per-service recovery rate $r$ and $T$ is the current CMHC services provision rate; note that as the current services provision rate, $T$, increases relative to the reference capacity $K$, the per-service recovery rate, ${rK}/T$, declines (due to increased pressure on services). CMHC services capacity (i.e., $K$) is assumed to increase at a constant rate per year, estimated from services usage data published by the Australian Institute of Health and Welfare (2018b).

*Disengagement* — Patients waiting for a GP, psychiatrist and allied health services, or psychiatric hospital care are assumed to disengage from the mental health care system at a constant per capita rate per year (estimated from data reported in Tyrer et al., 1995). The total disengagement rate therefore increases whenever the demand for mental health services exceeds services capacity, as the number of patients waiting for care continues to increase while patients are being referred to services at a higher rate than they can be treated. Patients receiving treatment also disengage from services due to dissatisfaction with the care provided (figures S16, S18−S19). Disengagement is assumed to increase the age-specific per capita rates at which people with moderate psychological distress transition to a state of high to very high psychological distress, due to a loss of hope that effective treatment is available, or trauma associated with unsatisfactory care (see Australian Bureau of Statistics, 2012a). Patients who have disengaged from the mental health services system return to the arrayed stock of people perceiving a need for care (i.e., they consider engaging with services again) at a constant per capita rate per year (figure S20).


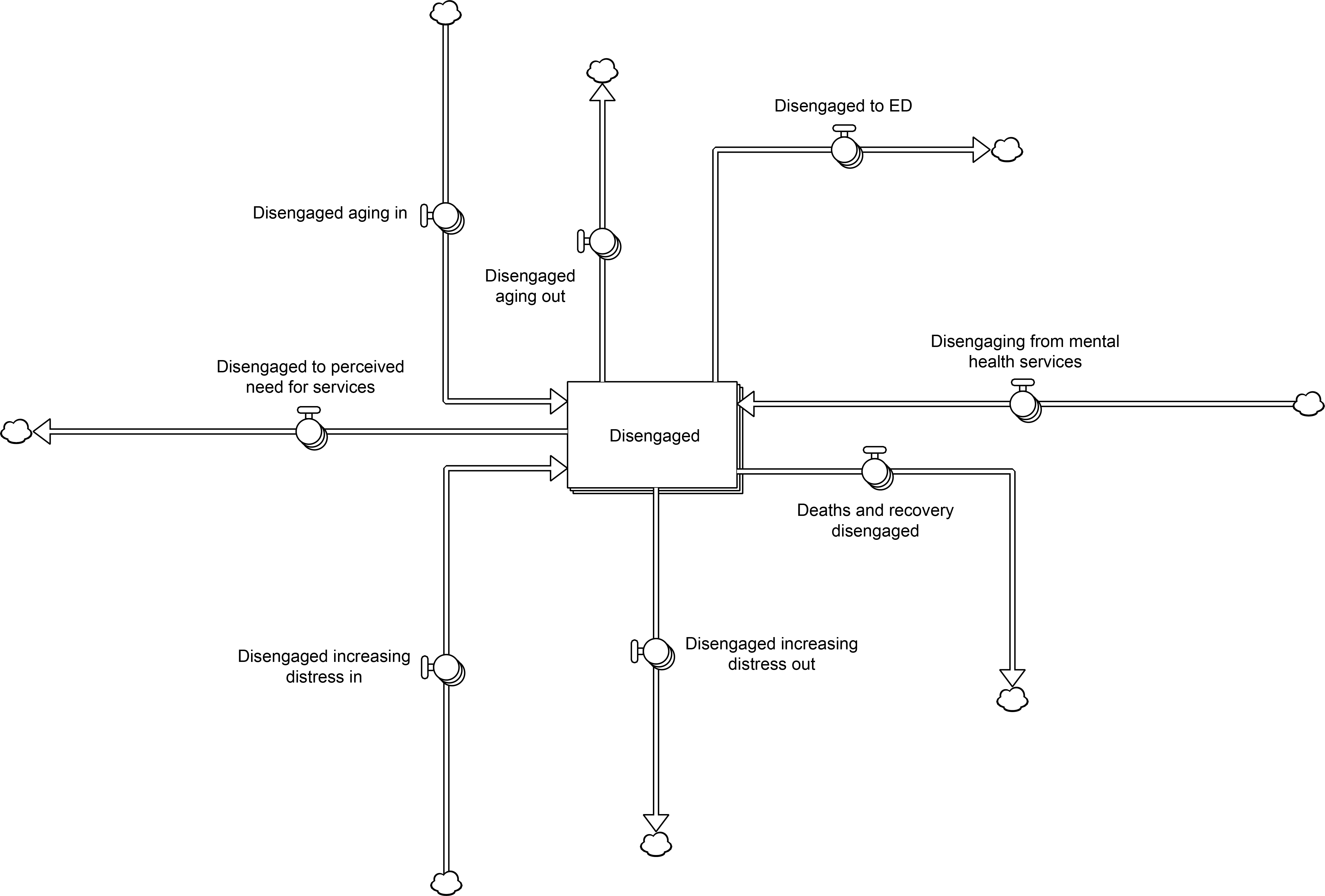


Figure S20. Stock and flow structure of the disengagement component of the mental health services sector.

*1.9. Suicidal behaviour sector*

Figure S21 presents the structure of the suicidal behaviour sector, which captures self-harm hospitalisations and suicide deaths (note that we equate suicide attempts with self-harm hospitalisations due to data availability constraints; see the Discussion section of the paper). Age-specific suicide attempt rates are calculated as $s_{i}\left( M_{i}-C_{i} \right)+\theta s_{i}\left( H_{i}-K_{i} \right)$, where $M_{i}$ and $H_{i}$ are the numbers of people in age group $i$ experiencing moderate psychological distress and high to very high psychological distress, respectively, $C_{i}$ and $K_{i}$ are the numbers of people in age group $i$ with moderate distress and high to very high distress receiving clinical care, $s_{i}$ is the per capita suicide attempt rate for moderately distressed people in age group $i$, and the attempt rate ratio $\theta$ is assumed to be greater than 1 (i.e., the per capita attempt rate for people with moderate distress is assumed to be less than that for people experiencing high to very high distress; Chamberlain et al., 2009). The number of suicide deaths per year is calculated as $\lambda a$, where $a$ is the suicide attempt rate and $\lambda$ is attempt lethality (i.e., the proportion of suicide attempts that are fatal, assumed to be constant). Self-harm hospitalisation and suicide death rate estimates derived from the model and from HealthStats NSW (http://www.healthstats.nsw.gov.au/) are presented in figure S22.


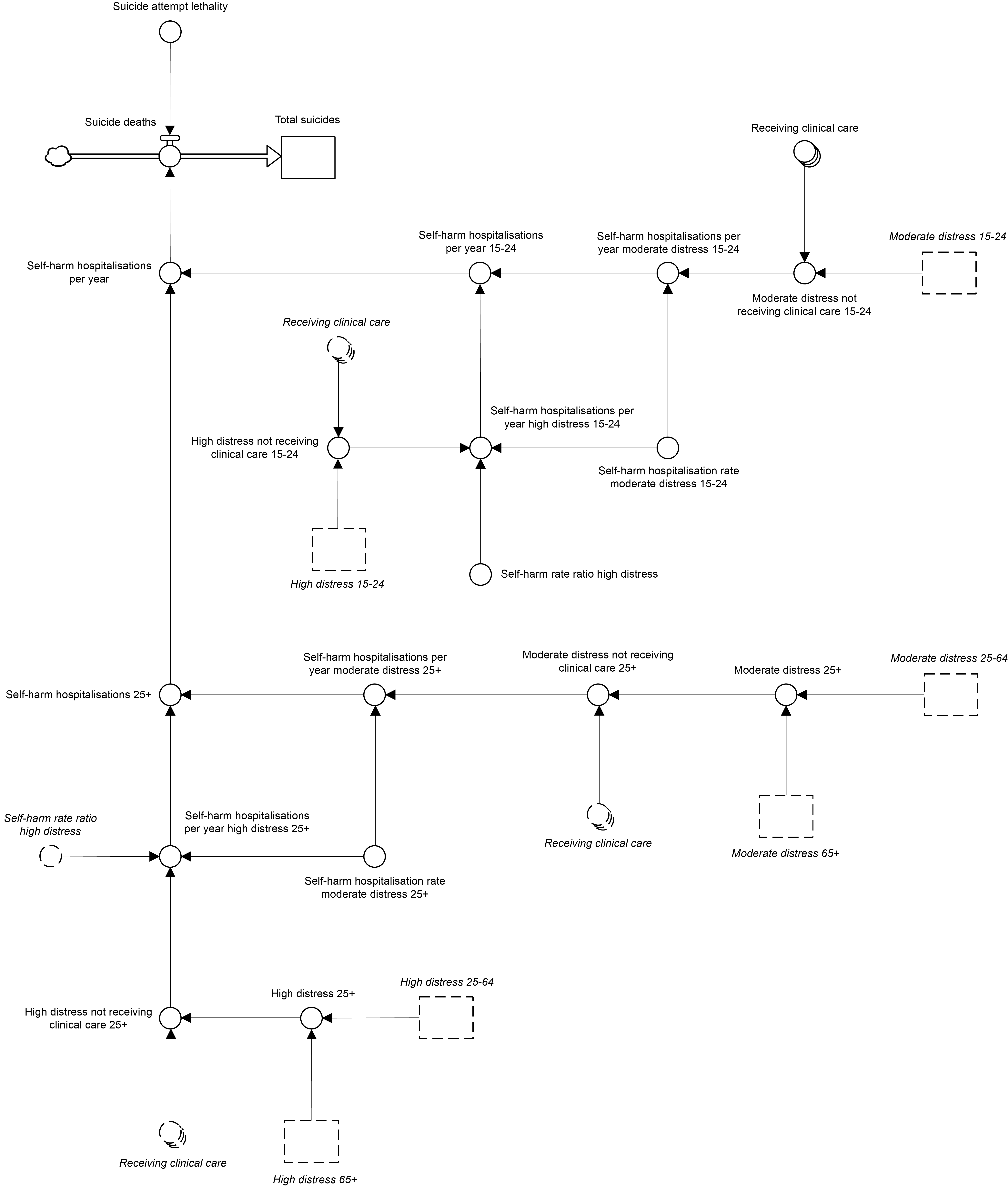


Figure S21. Structure of the suicidal behaviour sector.


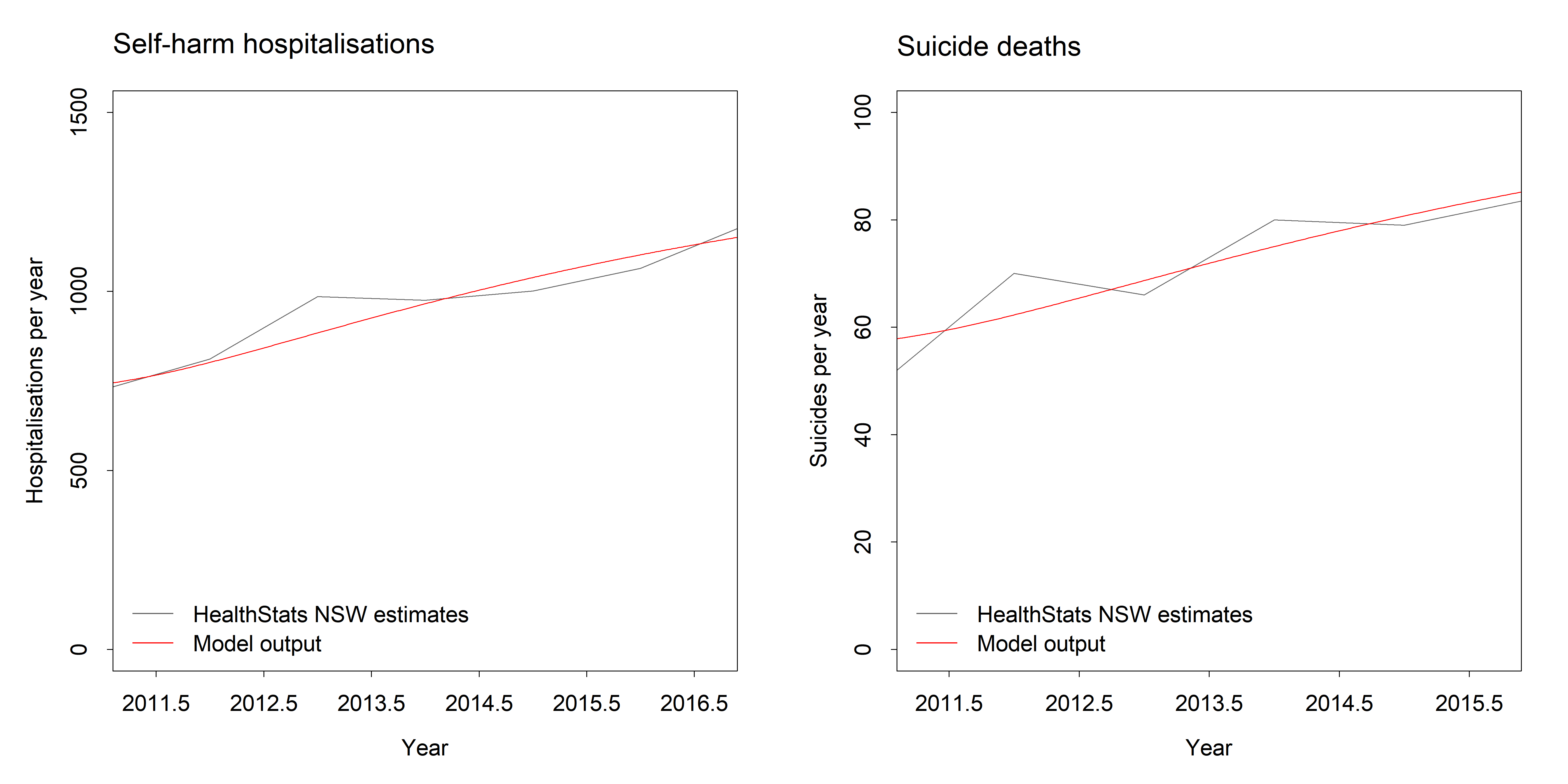


Figure S22. Self-harm hospitalisation and suicide death rate estimates derived from the system dynamics model and from HealthStats NSW (http://www.healthstats.nsw.gov.au/).

References

Australian Bureau of Statistics, 2012a. Information paper. Use of the Kessler psychological distress scale in ABS health surveys, Australia, 2007-08. Cat. no. 4817.0.55.001. Australian Bureau of Statistics, Canberra.

Australian Bureau of Statistics, 2012b. Information paper − A statistical definition of homelessness, Australia. Cat. no. 4922.0. Australian Bureau of Statistics, Canberra.

Australian Bureau of Statistics, 2015. National Health Survey: first results, 2014-15. Cat. no. 4364.0.55.001. Australian Bureau of Statistics, Canberra.

Australian Bureau of Statistics, 2016. Mental health and experiences of homelessness, Australia, 2014. Cat. no. 4329.0.00.005. Australian Bureau of Statistics, Canberra.

Australian Bureau of Statistics, 2017. Personal safety, Australia, 2016. Cat. no. 4906.0. Australian Bureau of Statistics, Canberra.

Australian Bureau of Statistics, 2018. Census of Population and Housing: Estimating homelessness, 2016. Cat. no. 2049.0. Australian Bureau of Statistics, Canberra.

Australian Bureau of Statistics, 2019a. Labour force, Australia, Sep 2019. Cat. no. 6202.0. Australian Bureau of Statistics, Canberra.

Australian Bureau of Statistics, 2019b. Recorded crime – victims, Australia, 2018. Cat. no. 4510.0. Australian Bureau of Statistics, Canberra.

Australian Institute of Health and Welfare, 2018a. Alcohol and other drug treatment services in Australia: 2016−17. Drug treatment series no. 31. Cat. no. HSE 207. Australian Institute of Health and Welfare, Canberra.

Australian Institute of Health and Welfare, 2018b. Mental health services − in brief 2018. Cat. no. HSE 211. Australian Institute of Health and Welfare, Canberra.

Björkenstam, E., Burström, B., Brännström, L., Vinnerljung, B, Björkenstam, C., Pebley, A. R., 2015. Cumulative exposure to childhood stressors and subsequent psychological distress. An analysis of US panel data. Soc. Sci. Med. 142, 109−117.

Chamberlain, P., Goldney, R., Delfabbro, P., Gill, T., Dal Grande, L., 2009. Suicidal ideation. The clinical utility of the K10. Crisis 30, 39−42.

Christensen, H., Griffiths, K. M., Jorm, A. F., 2004. Delivering interventions for depression by using the internet: randomised controlled trial. Br. Med. J. 328, 265.

Crum, R. M., Storr, C. L., Ialongo, N., Anthony, J. C., 2008. Is depressed mood in childhood associated with an increased risk for initiation of alcohol use during early adolescence? Addict. Behav. 33, 24−40.

Cuijpers, P., van Straten, A., van Schaik A., Andersson, G., 2009. Psychological treatment of depression in primary care: a meta-analysis. Br. J. Gen. Pract., doi: 10.3399/bjgp09X395139.

Frijters, P., Johnston, D. W., Shields, M. A., 2014. The effect of mental health on employment: evidence from Australian panel data. Health Econ. 23, 1058−1071.

Herman, D. B., Susser, E. S., Struening, E. L., Link, B. L., 1997. Adverse childhood experiences: are they risk factors for adult homelessness? Am. J. Public Health 87, 249−255.

Johnson, T. P., Freels, S. A., Parsons, J. A., Vangeest, J. B., 1997. Substance abuse and homelessness: social selection or social adaptation? Addiction 92, 437−445.

Kernic, M. A., Wolf, M. E., Holt, V. L., McKnight, B., Huebner, C. E., Rivara, F. P., 2003. Behavioral problems among children whose mothers are abused by an intimate partner. Child Abuse Negl. 27, 1231−1246.

Kim, D. R., Bale, T. L., Epperson, N., 2015. Prenatal programming of mental illness: current understanding of relationship and mechanism. Curr. Psychiatry Rep. 17, 5.

Kyriacou, D. N., Anglin, D., Taliaferro, E., Stone, S., Tubb, T., Linden, J. A., Muelleman, R., Barton, E., Kraus, J. F., 1999. Risk factors for injury to women from domestic violence. N. Engl. J. Med. 341, 1892−1898.

Lawrence, D., Johnson, S., Hafekost, J., Boterhoven de Haan, K., Sawyer, M., Ainley, J., Zubrick, S. R., 2015. The mental health of children and adolescents. Report on the second Australian Child and Adolescent Survey of Mental Health and Wellbeing. Department of Health, Canberra.

Lynskey, M. T., Fergusson, D. M., Horwood, L. J., 1994. The effect of parental alcohol problems on rates of adolescent psychiatric disorders. Addiction 89, 1277−1286.

Marmorstein, N. R., Iacono, W. G., Malone, S. M., 2010. Longitudinal associations between depression and substance dependence from adolescence through early adulthood. Drug Alcohol Depend. 107, 154−160.

Miller, W. R., Walters, S. T., Bennett, M. E., 2001. How effective in alcoholism treatment in the United States? J. Stud. Alcohol 62, 211−220.

Mitchell, W., Wray, L. R., Watts, M., 2019. Macroeconomics. Red Globe Press, London.

Morrison, D. S., 2009. Homelessness as an independent risk factor for mortality: results from a retrospective cohort study. Int. J. Epidemiol. 38, 877−883.

Ouellet-Morin, I., Fisher, H. L., York-Smith, M., Fincham-Campbell, S., Moffitt, T. E., Arseneault, L., 2015. Intimate partner violence and new-onset depression: a longitudinal study of women’s childhood and adult histories of abuse. Depress. Anxiety 32, 316−324.

Roerecke, M., Rehm, J., 2013. Alcohol use disorders and mortality: a systematic review and meta-analysis. Addiction 108, 1562−1578.

Russ, T. C., Stamatakis, E., Hamer, M., Starr, J. M., Kivimäki, M., Batty, G. D., 2012. Association between psychological distress and mortality: individual participant pooled analysis of 10 prospective cohort studies. Br. Med. J. 345, e4933.

Shelton, K. H., Taylor, P. J., Bonner, A., van den Bree, M., 2009. Risk factors for homelessness: evidence from a population-based study. Psychiatr. Serv. 60, 465−472.

Sorlie, P. D., Rogot, E., 1990. Mortality by employment status in the National Longitudinal Mortality Study. Am. J. Epidemiol. 132, 983−992.

Spinhoven, P., Elzinga, B. M., Van Hemert, A. M., de Rooij, M., Penninx, B. W., 2016. Childhood maltreatment, maladaptive personality types and level and course of psychological distress: A six-year longitudinal study. J. Affect. Disord. 191, 100−108.

Stevens, A., Radcliffe, P., Sanders, M., Hunt, N., 2008. Early exit: estimating and explaining early exit from drug treatment. Harm Reduct. J. 5, 13.

Tyrer, P., Morgan, J., Van Horn, E., Jayakody, M., Evans, K., Brummell, R., White, T., Baldwin, D., Harrison-Read, P., Johnson, T., 1995. A randomised controlled study of close monitoring of vulnerable psychiatric patients. Lancet 345, 756−759.

Weissman, M. M., Warner, V., Wickramaratne, P., Moreau, D., Olfson, M., 1997. Offspring of depressed parents. Arch. Gen. Psychiatry 54, 932−940.

Zabkiewicz, D., Schmidt, L. A., 2007. Behavioral health problems as barriers to work: results from a 6-year panel study of welfare recipients. J. Behav. Health Serv. Res. 34, 168−185.

2. Numerical inputs and data sources

Table S1. Numerical inputs and data sources. Inputs highlighted in red were varied in the sensitivity analyses (see Methods section of the paper).

| Input label | Value(s) | Notes |
| --- | --- | --- |
|  |  |  |
| Population sector |  |  |
|  |  |  |
| Birth rate increase per year | -0.005121411 | Fractional rate of increase per year. Estimated via constrained optimisation |
| Birth rate per year initial | 0.00983585 | Per capita birth rate per year. Estimated via constrained optimisation. |
| Death rate increase per year | -0.033013218 | Fractional rate of increase per year. Estimated via constrained optimisation |
| Death rate per year initial | 0.007641254 | Per capita mortality rate per year. Estimated via constrained optimisation. |
| Death rate ratio | 0.056828792 (0-14 years), 0.055599989 (15-24 years), 0.316053003 (25-64 years), 5.765317106 (65+ years) | Age-specific per capita mortality per year divided by total per capita mortality per year. Mortality rates were estimated using state-level data obtained from HealthStats NSW (see http://www.healthstats.nsw.gov.au/). |
| Emigration rate | 0.002736476 (0-14 years), 0.040969955 (15-24 years), 0.000674608 (25-64 years), 0.006956509 (65+ years | Per capita emigration rate per year. Estimated via constrained optimisation. |
| Immigration rate | 39.68586628 (0-14 years), 21.72432524 (15-24 years), 41.50331318 (25-64 years), 43.9723603 (65+ years) | Number of people immigrating per year. Estimated via constrained optimisation. |
| Proportion female | 0.486986489 (0-14 years), 0.479938453 (15-24 years), 0.513144357 (25-64 years), 0.532801136 (65+ years) | Derived from population estimates and projections for the period 2001-2036 obtained from HealthStats NSW (see http://www.healthstats.nsw.gov.au/) |
|  |  |  |
| Psychological distress sector |  |  |
|  |  |  |
| Death rate ratio high distress | 1.37 | Derived from Russ et al. (2012, Br. Med. J. 345, e4933) |
| Death rate ratio moderate distress | 1.16 | Derived from Russ et al. (2012, Br. Med. J. 345, e4933) |
| Domestic violence rate ratio low-moderate distress | 0.88116633 | Domestic violence rate among people aged 15 years and above with low or moderate psychological distress divided by the domestic violence rate in the total population age 15 years and above. Rate estimates were derived from Kazantzis et al. (2000, N. Z. J. Psychol. 29, 67−73). |
| Effect of domestic violence on psychological distress | 1.72 | Derived from Ouellet-Morin et al. (2015, Depress. Anxiety 32, 316−324) |
| Effect of high risk of mental disorder on psychological distress | 2.283777 | Derived from Björkenstam et al. (2015, Soc. Sci. Med. 142, 109−117) |
| Effect of homelessness on psychological distress | 4.466664 | Derived from Australian Bureau of Statistics (2012, Information paper. Use of the Kessler psychological distress scale in ABS health surveys, Australia, 2007-08. Cat. no. 4817.0.55.001. Australian Bureau of Statistics, Canberra) |
| Effect of moderate risk of mental disorder on psychological distress | 1.523867 | Derived from Björkenstam et al. (2015, Soc. Sci. Med. 142, 109−117) |
| Effect of substance abuse on psychological distress | 2.63 | Derived from Marmorstein et al. (2010, Drug Alcohol Depend. 107, 154−160) |
| Effect of unemployment on low to moderate distress | 1.26294 | Derived from Australian Bureau of Statistics (2012, Information paper. Use of the Kessler psychological distress scale in ABS health surveys, Australia, 2007-08. Cat. no. 4817.0.55.001. Australian Bureau of Statistics, Canberra) |
| Effect of unemployment on moderate to high distress | 2.637782 | Derived from Australian Bureau of Statistics (2012, Information paper. Use of the Kessler psychological distress scale in ABS health surveys, Australia, 2007-08. Cat. no. 4817.0.55.001. Australian Bureau of Statistics, Canberra) |
| High risk prevalence ratio low distress | 0.8735998 | Prevalence of high mental disorder risk among people with low psychological distress divided by the prevalence of high mental disorder risk in the total population. Prevalence estimates were derived from Björkenstam et al. (2015, Soc. Sci. Med. 142, 109−117). |
| High risk prevalence ratio moderate distress | 1.091343 | Prevalence of high mental disorder risk among people with moderate psychological distress divided by the prevalence of high mental disorder risk in the total population. Prevalence estimates were derived from Björkenstam et al. (2015, Soc. Sci. Med. 142, 109−117). |
| High to moderate distress rate | 0.250094441 | Per capita rate per year. Estimated via constrained optimisation. |
| Homelessness prevalence ratio low distress | 0.5276654 | Prevalence of homelessness among people with low psychological distress divided by the prevalence of homelessness in the total population. Prevalence estimates were derived from Australian Bureau of Statistics (2012, Information paper. Use of the Kessler psychological distress scale in ABS health surveys, Australia, 2007-08. Cat. no. 4817.0.55.001. Australian Bureau of Statistics, Canberra). |
| Homelessness prevalence ratio moderate distress | 1.259076 | Prevalence of homelessness among people with moderate psychological distress divided by the prevalence of homelessness in the total population. Prevalence estimates were derived from Australian Bureau of Statistics (2012, Information paper. Use of the Kessler psychological distress scale in ABS health surveys, Australia, 2007-08. Cat. no. 4817.0.55.001. Australian Bureau of Statistics, Canberra). |
| Low to moderate distress base rate | 0.0740892689742 (15-24 years), 0.0535941486815 (25-64 years), 0.0192150630406 (65+ years) | Per capita rate per year. Estimated via constrained optimisation. |
| Migrant high distress prevalence | 0.144125 (15-24 years), 0.126872357 (25-64 years), 0.075526047 (65+ years) | State-level estimates of high to very high psychological distress prevalence derived from HealthStats NSW (see http://www.healthstats.nsw.gov.au/) |
| Migrant low distress prevalence | 0.58725 (15-24 years), 0.662830909 (25-64 years), 0.764079908 (65+ years) | State-level estimates of low psychological distress prevalence derived from HealthStats NSW (see http://www.healthstats.nsw.gov.au/) |
| Migrant moderate distress prevalence | 0.268625 (15-24 years), 0.210296735 (25-64 years), 0.160394045 (65+ years) | State-level estimates of moderate psychological distress prevalence derived from HealthStats NSW (see http://www.healthstats.nsw.gov.au/) |
| Moderate risk prevalence ratio low distress | 0.9326939 | Prevalence of moderate mental disorder risk among people with low psychological distress divided by the prevalence of moderate mental disorder risk in the total population. Prevalence estimates were derived from Björkenstam et al. (2015, Soc. Sci. Med. 142, 109−117). |
| Moderate risk prevalence ratio moderate distress | 1.113829 | Prevalence of moderate mental disorder risk among people with moderate psychological distress divided by the prevalence of moderate mental disorder risk in the total population. Prevalence estimates were derived from Björkenstam et al. (2015, Soc. Sci. Med. 142, 109−117). |
| Moderate to high distress base rate | 0.162523120501 (15-24 years), 0.21475648368 (25-64 years), 0.159545397942 (65+ years) | Per capita rate per year. Estimated via constrained optimisation. |
| Moderate to low distress rate | 0 | Per capita rate per year. Estimated via constrained optimisation. |
| Substance abuse prevalence ratio low distress | 0.6595638 | Prevalence of substance abuse among people with low psychological distress divided by the prevalence of substance abuse in the total population. Prevalence estimates were derived from Australian Bureau of Statistics (2012, Information paper. Use of the Kessler psychological distress scale in ABS health surveys, Australia, 2007-08. Cat. no. 4817.0.55.001. Australian Bureau of Statistics, Canberra). |
| Substance abuse prevalence ratio moderate distress | 1.392881 | Prevalence of substance abuse among people with moderate psychological distress divided by the prevalence of substance abuse in the total population. Prevalence estimates were derived from Australian Bureau of Statistics (2012, Information paper. Use of the Kessler psychological distress scale in ABS health surveys, Australia, 2007-08. Cat. no. 4817.0.55.001. Australian Bureau of Statistics, Canberra). |
| Unemployment rate ratio low distress | 0.6961765 | Unemployment rate among people with low psychological distress divided by the total unemployment rate. Unemployment rate estimates were derived from Australian Bureau of Statistics (2012, Information paper. Use of the Kessler psychological distress scale in ABS health surveys, Australia, 2007-08. Cat. no. 4817.0.55.001. Australian Bureau of Statistics, Canberra). |
| Unemployment rate ratio moderate distress | 1.233869 | Unemployment rate among people with moderate psychological distress divided by the total unemployment rate. Unemployment rate estimates were derived from Australian Bureau of Statistics (2012, Information paper. Use of the Kessler psychological distress scale in ABS health surveys, Australia, 2007-08. Cat. no. 4817.0.55.001. Australian Bureau of Statistics, Canberra). |
|  |  |  |
| Early life sector |  |  |
|  |  |  |
| Childhood abuse rate ratio low risk 0-14 | 0.6347065 | Abuse rate among children aged 0-14 years at low risk of developing a mental disorder divided by the total abuse rate for children aged 0-14 years. Estimates of abuse rates were derived from Spinhoven et al. (2016, J. Affect. Disord. 191, 100−108). |
| Childhood abuse rate ratio moderate risk 0-14 | 1.121226 | Abuse rate among children aged 0-14 years at moderate risk of developing a mental disorder divided by the total abuse rate for children aged 0-14 years. Estimates of abuse rates were derived from Spinhoven et al. (2016, J. Affect. Disord. 191, 100−108). |
| Domestic violence base rate increase per year 0-14 | -6.52455E-05 | Estimated via constrained optimisation |
| Domestic violence base rate per year initial 0-14 | 0.002445339 | Estimated via constrained optimisation |
| Domestic violence exposure rate ratio low risk 0-14 | 0.6347065 | Domestic violence exposure rate among children aged 0-14 years at low risk of developing a mental disorder divided by the total domestic violence exposure rate for children aged 0-14 years. Estimates of abuse rates were derived from Spinhoven et al. (2016, J. Affect. Disord. 191, 100−108). |
| Domestic violence exposure rate ratio moderate risk 0-14 | 1.121226 | Domestic violence exposure rate among children aged 0-14 years at moderate risk of developing a mental disorder divided by the total domestic violence exposure rate for children aged 0-14 years. Estimates of abuse rates were derived from Spinhoven et al. (2016, J. Affect. Disord. 191, 100−108). |
| Effect of childhood abuse on risk of mental disorder 0-14 | 2.594792 | Derived from Spinhoven et al. (2016, J. Affect. Disord. 191, 100−108) |
| Effect of domestic violence exposure on risk of mental disorder 0-14 | 1.4 | Derived from Kernic et al. (2003, Child Abuse Negl. 27, 1231−1246) |
| Effect of high parental distress on risk of mental disorder 0-14 | 2.39 | Derived from Weissman et al. (1997, Arch. Gen. Psychiatry 54, 932−940) |
| Effect of parental substance abuse on risk of mental disorder 0-14 | 2.783154 | Derived from Lynskey et al. (1994, Addiction 89, 1277−1286) |
| Low to moderate risk of mental disorder base rate 0-14 | 0.026404445 | Per capita rate per year. Estimated via constrained optimisation. |
| Mean children per family | 1.833692857 | Derived from Australian Bureau of Statistics (2017, Data by region, 2011-16. Cat. no. 1410.0. Australian Bureau of Statistics, Canberra) |
| Migrant high risk prevalence | 0.1556 | State-level estimate of high to very high psychological distress prevalence among children aged 12-17 years calculated using data from HealthStats NSW (see http://www.healthstats.nsw.gov.au/) |
| Migrant low risk prevalence | 0.616864322 | State-level estimate of low psychological distress prevalence among children aged 12-17 years calculated using data from HealthStats NSW (see http://www.healthstats.nsw.gov.au/) and Lawrence et al. (2015, The mental health of children and adolescents. Report on the second Australian Child and Adolescent Survey of Mental Health and Wellbeing. Department of Health, Canberra) |
| Migrant moderate risk prevalence | 0.227535678 | State-level estimate of moderate psychological distress prevalence among children aged 12-17 years calculated using data from HealthStats NSW (see http://www.healthstats.nsw.gov.au/) and Lawrence et al. (2015, The mental health of children and adolescents. Report on the second Australian Child and Adolescent Survey of Mental Health and Wellbeing. Department of Health, Canberra) |
| Moderate to high risk of mental disorder base rate 0-14 | 0.031230955 | Per capita rate per year. Estimated via constrained optimisation. |
| Parental distress prevalence ratio low risk 0-14 | 0.7900775 | Parental psychological distress prevalence among children aged 0-14 years at low risk of developing a mental disorder divided by the total prevalence of parental psychological distress. Estimates of parental psychological distress prevalence were derived from Weissman et al. (1997, Arch. Gen. Psychiatry 54, 932−940). |
| Parental distress prevalence ratio moderate risk 0-14 | 0.7900775 | Parental psychological distress prevalence among children aged 0-14 years at moderate risk of developing a mental disorder divided by the total prevalence of parental psychological distress. Estimates of parental psychological distress prevalence were derived from Weissman et al. (1997, Arch. Gen. Psychiatry 54, 932−940). |
| Parental substance abuse rate ratio low risk 0-14 | 0.8047011 | Parental substance abuse prevalence among children aged 0-14 years at low risk of developing a mental disorder divided by the total prevalence of parental substance abuse. Estimates of parental substance abuse prevalence were derived from Lynskey et al. (1994, Addiction 89, 1277−1286). |
| Parental substance abuse rate ratio moderate risk 0-14 | 0.8047011 | Parental substance abuse prevalence among children aged 0-14 years at moderate risk of developing a mental disorder divided by the total prevalence of parental substance abuse. Estimates of parental substance abuse prevalence were derived from Lynskey et al. (1994, Addiction 89, 1277−1286). |
| Proportion with dependent children females 15-24 | 0.032882886 | Derived from Australian Bureau of Statistics (2015, Family Characteristics and Transitions, Australia, 2012-13. Cat. no. 4442.0. Australian Bureau of Statistics, Canberra) |
| Proportion with dependent children females 25-64 | 0.277982526 | Derived from Australian Bureau of Statistics (2015, Family Characteristics and Transitions, Australia, 2012-13. Cat. no. 4442.0. Australian Bureau of Statistics, Canberra) |
| Proportion with dependent children females 65+ | 0.017383636 | Derived from Australian Bureau of Statistics (2015, Family Characteristics and Transitions, Australia, 2012-13. Cat. no. 4442.0. Australian Bureau of Statistics, Canberra) |
|  |  |  |
| Employment sector |  |  |
|  |  |  |
| Death rate ratio unemployed | 1.22 | Derived from Sorlie and Rogot (1990, Am. J. Epidemiol. 132, 983−992) |
| Effect of high distress on employment | 0.6944646 | Derived from Frijters et al. (2014, Health Econ. 23, 1058−1071) |
| Effect of moderate distress on employment | 0.8874491 | Derived from Frijters et al. (2014, Health Econ. 23, 1058−1071) |
| Effect of substance abuse on employment | 0.31 | Derived from Zabkiewicz and Schmidt (2007, J. Behav. Health Serv. Res. 34, 168−185) |
| Employed to NILF base rate | 0.000104854 | Per capita rate per year. Estimated via constrained optimisation. |
| Employed to NILF rate ratio | 2.10170439709 (25-64 years), 4414.55728601 (65+ years) | Estimated via constrained optimisation |
| Employed to unemployed base rate | 0.011875453 | Per capita rate per year. Estimated via constrained optimisation. |
| High distress prevalence ratio unemployed | 2.308333 | Prevalence of high to very high psychological distress in the unemployed population divided by the prevalence of high to very high psychological distress in the total population. Prevalence estimates were derived from Australian Bureau of Statistics (2012, Information paper. Use of the Kessler psychological distress scale in ABS health surveys, Australia, 2007-08. Cat. no. 4817.0.55.001. Australian Bureau of Statistics, Canberra). |
| Migrant employed fraction | 0.580750097 (15-24 years), 0.755277453 (25-64 years), 0.119746234 (65+ years) | State-level estimates of the proportion of people employed derived from Australian Bureau of Statistics (2019, Labour force, Australia, Sep 2019. Cat. no. 6202.0. Australian Bureau of Statistics, Canberra) |
| Migrant unemployed fraction | 0.074720831 (15-24 years), 0.032017055 (25-64 years), 0.001647848 (65+ years) | State-level estimates of the proportion of people unemployed derived from Australian Bureau of Statistics (2019, Labour force, Australia, Sep 2019. Cat. no. 6202.0. Australian Bureau of Statistics, Canberra) |
| Moderate distress prevalence ratio unemployed | 1.231884 | Prevalence of moderate psychological distress in the unemployed population divided by the prevalence of moderate psychological distress in the total population. Prevalence estimates were derived from Australian Bureau of Statistics (2012, Information paper. Use of the Kessler psychological distress scale in ABS health surveys, Australia, 2007-08. Cat. no. 4817.0.55.001. Australian Bureau of Statistics, Canberra). |
| NILF to unemployed rate ratio | 0.0761187382775 (25-64 years), 0 (65+ years) | Estimated via constrained optimisation |
| NILF to unemployed base rate | 0.374746165 | Per capita rate per year. Estimated via constrained optimisation. |
| Substance abuse prevalence ratio unemployed | 2.176471 | Prevalence of substance abuse in the unemployed population divided by the prevalence of substance abuse in the total population. Prevalence estimates were derived from Slade et al. (2009, The Mental Health of Australians 2. Report on the 2007 National Survey of Mental Health and Wellbeing. Department of Health and Ageing, Canberra). |
| Unemployed to employed base rate | 1.388901382 | Per capita rate per year. Estimated via constrained optimisation. |
| Unemployed to employed rate ratio | 0.339106331534 (25-64 years), 0 (65+ years) | Estimated via constrained optimisation |
| Unemployed to NILF base rate | 0.047191656 | Per capita rate per year. Estimated via constrained optimisation. |
| Unemployed to NILF rate coefficient unemployed | 1.129277923 | Effect of the unemployment rate on labour force participation. Estimated via constrained optimisation. |
| Unemployed to NILF rate ratio 65+ | 69.13251807 | Estimated via constrained optimisation |
|  |  |  |
| Substance abuse sector |  |  |
|  |  |  |
| AOD treatment services capacity increase per year | 76.37779448 | Annual increase in the total number of closed treatment episodes commencing per week. The default value was estimated using PHN-level treatment services data for 2014–2017 published by the Australian Institute of Health and Welfare (available at: https://www.aihw.gov.au/reports/alcohol-other-drug-treatment-services/aodts-2016-17/data) assuming services were operating at (near-) maximum capacity over this period. |
| AOD treatment services capacity initial | 4471.150612 | The initial number of closed treatment episodes commencing per week (i.e., at the start of 2011). Estimated via constrained optimisation. |
| Death rate ratio substance abuse disorder | 1.95 | Derived from Roerecke and Rehm (2013, Addiction 108, 1562−1578) |
| Disengagement rate AOD treatment services | 0.05434219 | Derived from Stevens et al. (2008, Harm Reduct. J. 5, 13) |
| Effect of high distress on substance abuse | 3.035 | Derived from Marmorstein et al. (2010, Drug Alcohol Depend. 107, 154−160) |
| Effect of high risk of mental disorder on substance abuse | 1.62 | Derived from Crum et al. (2008, Addict. Behav. 33, 24−40) |
| Effect of homelessness on substance abuse | 1.65 | Derived from Johnson et al. (1997, Addiction 92, 437−445) |
| Effect of moderate risk of mental disorder on substance abuse | 1.08 | Derived from Crum et al. (2008, Addict. Behav. 33, 24−40) |
| High distress prevalence ratio no substance abuse | 0.8137255 | Prevalence of high to very high psychological distress among people without a substance abuse disorder divided by the prevalence of high to very high psychological distress in the total population. Prevalence estimates were derived from Marmorstein et al. (2010, Drug Alcohol Depend. 107, 154−160). |
| High risk prevalence ratio no substance abuse | 0.8609731 | Prevalence of high mental disorder risk among people without a substance abuse disorder divided by the prevalence of high mental disorder risk in the total population. Prevalence estimates were derived from Crum et al. (2008, Addict. Behav. 33, 24−40). |
| Homelessness prevalence ratio no substance abuse | 0.8683555 | Prevalence of homelessness among people without a substance abuse disorder divided by the prevalence of homelessness in the total population. Prevalence estimates were derived from Slade et al. (2009, The Mental Health of Australians 2. Report on the 2007 National Survey of Mental Health and Wellbeing. Department of Health and Ageing, Canberra). |
| Mean AOD treatment duration (weeks) | 2.028571429 | Derived from state-level treatment services data published by the Australian Institute of Health and Welfare (available at: https://www.aihw.gov.au/reports/alcohol-other-drug-treatment-services/aodts-2016-17/data) |
| Migrant substance abuse disorder fraction | 0.109891368 (15-24 years), 0.072721461 (25+ years) | State-level estimates of substance abuse disorder prevalence calculated using data from HealthStats NSW (see http://www.healthstats.nsw.gov.au/) and Australian Bureau of Statistics (2015, National Health Survey: first results, 2014-15. Cat. no. 4364.0.55.001. Australian Bureau of Statistics, Canberra) |
| Moderate risk prevalence ratio no substance abuse | 1.003867 | Prevalence of moderate mental disorder risk among people without a substance abuse disorder divided by the prevalence of moderate mental disorder risk in the total population. Prevalence estimates were derived from Crum et al. (2008, Addict. Behav. 33, 24−40). |
| Referral rate AOD treatment services | 0.400701017156 (15-24 years), 0.17482470934 (25+ years) | Per capita rate per year. Estimated via constrained optimisation. |
| Substance abuse disorder onset base rate | 0.0120146677783 (15-24 years), 0.000597091359846 (25+ years) | Per capita rate per year. Estimated via constrained optimisation. |
| Substance abuse disorder relapse fraction | 0.759 | Derived from Miller et al. (2001, J. Stud. Alcohol 62, 211−220) |
|  |  |  |
| Domestic violence and homelessness sectors |  |  |
|  |  |  |
| Death rate ratio homeless | 1.6 | Derived from Morrison (2009, Int. J. Epidemiol. 38, 877−883) |
| Domestic violence base rate increase per year | -0.000159018237307 (15-24 years), -0.0000405600664465 (25-64 years), 0.0000151487030917 (65+ years) | Estimated via constrained optimisation |
| Domestic violence base rate per year initial | 0.0112217974325 (15-24 years), 0.00600842083676 (25-64 years), 0.000425480905761 (65+ years) | Estimated via constrained optimisation |
| Effect of high risk of mental disorder on homelessness | 7.8 | Derived from Herman et al. (1997, Am. J. Public Health 87, 249−255) |
| Effect of partner substance abuse on domestic violence rate | 3.6 | Derived from Kyriacou et al. (1999, N. Engl. J. Med. 341, 1892−1898) |
| Effect of partner unemployment on domestic violence rate | 2.4 | Derived from Kyriacou et al. (1999, N. Engl. J. Med. 341, 1892−1898) |
| Effect of substance abuse on homelessness | 1.16 | Derived from Shelton et al. (2009, Psychiatr. Serv. 60, 465−472) |
| Entering homelessness base rate | 0.00378779355484 (0-14 years), 0.0133130899957 (15-24 years), 0.0067217685284 (25-64 years), 0.00196039726167 (65+ years) | Per capita rate per year. Estimated via constrained optimisation. |
| Mean duration of homelessness (weeks) | 20.42345 | Derived from Australian Bureau of Statistics (2016, Mental health and experiences of homelessness, Australia, 2014. Cat. no. 4329.0.00.005. Australian Bureau of Statistics, Canberra) |
| Probability of entering homelessness per domestic violence incident | 0.023392 | Derived from Australian Bureau of Statistics (2017, Personal safety, Australia, 2016. Cat. no. 4906.0. Australian Bureau of Statistics, Canberra) |
|  |  |  |
| Mental health services sector |  |  |
|  |  |  |
| Additional admission rate non-specialised hospital care age array | 0 (15-24 years), 0.00946813178634 (25-64 years), 0.0254844007811 (65+ years) | Per capita rate per year. Estimated via constrained optimisation. |
| Additional CMHC service contacts rate age array | 1.69369513252 (15-24 years), 2.78950696051 (25-64 years), 0.468803128216 (65+ years) | Per capita rate per year. Estimated via constrained optimisation. |
| Additional psychiatrist or allied services rate age array | 3.1937593668 (15-24 years), 5.81567353336 (25-64 years), 1.32655473201 (65+ years) | Per capita rate per year. Estimated via constrained optimisation. |
| CMHC services capacity increase per year | 0 | Annual increase in the total number of community mental health care service contacts per week. The default value (corresponding to no capacity growth) was derived from service usage data for 2008–2017 published by the Australian Institute of Health and Welfare (available at: https://www.aihw.gov.au/reports-data/health-welfare-services/mental-health-services/data). For the sensitivity analyses, values were drawn from a uniform distribution spanning +/- 2% of initial capacity (as opposed to 20% of the default value of 0; see Methods section of the paper). |
| CMHC services capacity initial | 3757.164849 | The initial number of community mental health care service contacts per week (i.e., at the start of 2011). Estimated via constrained optimisation. |
| CMHC services referral rate ED | 0.402481693 | Fraction of patients discharged from emergency department care referred to community mental health care services. Estimated via constrained optimisation. |
| Disengaged to perceived need for services rate | 5.019914378 | Per capita rate per year. Estimated via constrained optimisation. |
| Disengagement rate hospital care | 0.048 | Derived from state-level consumer survey data for 2016-17 published by the Australian Institute of Health and Welfare (available at: https://www.aihw.gov.au/reports-data/health-welfare-services/mental-health-services/data) |
| Disengagement rate non-hospital care | 0.033 | Derived from state-level consumer survey data for 2016-17 published by the Australian Institute of Health and Welfare (available at: https://www.aihw.gov.au/reports-data/health-welfare-services/mental-health-services/data) |
| Disengagement rate waiting | 0.2620284 | Derived from Tyrer et al. (1995, Lancet 345, 756−759) |
| ED presentation base rate 65+ | 0.002402956 | Per capita rate per year. Estimated via constrained optimisation. |
| ED presentation rate ratio age array | 2.293531831 (15-24 years), 1.599009846 (25-64 years), 1 (65+ years) | Per capita rate ratios for people aged 15-24 years and 25-64 years were estimated via constrained optimisation |
| ED presentation rate ratio perceived need for services | 9.99750026 | Estimated via constrained optimisation |
| Effect of disengagement on psychological distress | 1.271517 | Derived from Australian Bureau of Statistics (2012, Information paper. Use of the Kessler psychological distress scale in ABS health surveys, Australia, 2007-08. Cat. no. 4817.0.55.001. Australian Bureau of Statistics, Canberra) |
| Effect of high distress on CMHC services referral rate GP | 3.790613 | Derived from Australian Bureau of Statistics (2012, Information paper. Use of the Kessler psychological distress scale in ABS health surveys, Australia, 2007-08. Cat. no. 4817.0.55.001. Australian Bureau of Statistics, Canberra) |
| Effect of high distress on ED presentation rate | 3.790613 | Derived from Australian Bureau of Statistics (2012, Information paper. Use of the Kessler psychological distress scale in ABS health surveys, Australia, 2007-08. Cat. no. 4817.0.55.001. Australian Bureau of Statistics, Canberra) |
| Effect of high distress on help seeking | 0.9858641 | Derived from Australian Bureau of Statistics (2012, Information paper. Use of the Kessler psychological distress scale in ABS health surveys, Australia, 2007-08. Cat. no. 4817.0.55.001. Australian Bureau of Statistics, Canberra) |
| Effect of high distress on non-specialised hospitalisation rate | 3.790613 | Derived from Australian Bureau of Statistics (2012, Information paper. Use of the Kessler psychological distress scale in ABS health surveys, Australia, 2007-08. Cat. no. 4817.0.55.001. Australian Bureau of Statistics, Canberra) |
| Effect of high distress on psychiatric hospital admission | 3.790613 | Derived from Australian Bureau of Statistics (2012, Information paper. Use of the Kessler psychological distress scale in ABS health surveys, Australia, 2007-08. Cat. no. 4817.0.55.001. Australian Bureau of Statistics, Canberra) |
| GP services capacity increase per year | 125.8463337 | Annual increase in the total number of mental health-related general practitioner consultations that can be completed per week. The default value was estimated using Medicare Benefits Schedule (MBS) data for 2012–2017 assuming services were operating at (near-) maximum capacity over this period. |
| GP services capacity initial | 1115.471912 | The initial number of mental health-related general practitioner consultations completed per week (i.e., at the start of 2011). Estimated via constrained optimisation. |
| Hospital admission rate increase rate | 0.003214091 | Estimated via constrained optimisation |
| Hospital admission rate initial | 0.35376657 | Initial fraction of patients presenting to an emergency department admitted to hospital (i.e., at the start of 2011). Estimated via constrained optimisation using national data on mental health-related emergency department presentations published by the Australian Institute of Health and Welfare (available at: https://www.aihw.gov.au/reports-data/health-welfare-services/mental-health-services/data). |
| Mean treatment duration non-specialised hospital care (weeks) | 0.8746473 | Derived from national data on mental health-related hospitalisations published by the Australian Institute of Health and Welfare (available at: https://www.aihw.gov.au/reports-data/health-welfare-services/mental-health-services/data) |
| Mean treatment duration online services (weeks) | 6 | Derived from Christensen et al. (2004, Br. Med. J. 328, 265) |
| Mean treatment duration psychiatric hospital (weeks) | 3.026475 | Derived from national data on mental health-related hospitalisations published by the Australian Institute of Health and Welfare (available at: https://www.aihw.gov.au/reports-data/health-welfare-services/mental-health-services/data) |
| Non-specialised hospital capacity increase per year | 4.227875997 | Annual increase in the maximum number of general hospital admissions for mental disorders per week. The default value was estimated using hospital separations data for 2011–2018 available from HealthStats NSW (http://www.healthstats.nsw.gov.au) and data on the provision of non-specialised mental health care in public hospitals published by the Australian Institute of Health and Welfare (available at: https://www.aihw.gov.au/reports-data/health-welfare-services/mental-health-services/data). |
| Non-specialised hospital capacity initial | 25.15440629 | The initial number of general hospital admissions for mental disorders per week (i.e., at the start of 2011). Estimated via constrained optimisation. |
| Non-specialised hospital care to GP services rate | 0.4 | Default value assumes that 40% of patients discharged from non-specialised hospital care who do not disengage from services are referred to a general practitioner |
| Non-specialised hospital care to psychiatrist or allied services rate | 0.1 | Default value assumes that 10% of patients discharged from non-specialised hospital care who do not disengage from services are referred to psychiatrist or allied health services |
| Perceiving need for services base rate | 0.340972378059 (moderate distress), 0.629840868527 (high distress) | Per capita rate per year. Estimated via constrained optimisation. |
| Perceiving need for services rate increase per year | 0.00508479 | Estimated via constrained optimisation |
| Psychiatric hospital admission proportion | 0.518727328 | Derived from national data on mental health-related hospitalisations published by the Australian Institute of Health and Welfare (available at: https://www.aihw.gov.au/reports-data/health-welfare-services/mental-health-services/data) |
| Psychiatric hospital capacity increase per year | 4.916764181 | Annual increase in the maximum number of psychiatric hospital admissions per week. The default value was estimated using hospital separations data for 2011–2018 available from HealthStats NSW (http://www.healthstats.nsw.gov.au) and data on the provision of specialised psychiatric care in public hospitals published by the Australian Institute of Health and Welfare (available at: https://www.aihw.gov.au/reports-data/health-welfare-services/mental-health-services/data). |
| Psychiatric hospital capacity initial | 29.24244478 | The initial number of psychiatric hospital admissions per week (i.e., at the start of 2011). Estimated via constrained optimisation. |
| Psychiatric hospital care to GP services rate | 0.11581282 | Fraction of patients. Estimated via constrained optimisation. |
| Psychiatric hospital care to psychiatrist or allied services rate | 0.212754316 | Fraction of patients. Estimated via constrained optimisation. |
| Psychiatrist and allied services capacity increase per year | 216.3105996 | Annual increase in the total number of psychiatrist and allied services that can be provided per week. The default value was estimated using MBS data for 2012–2017 assuming services were operating at (near-) maximum capacity over this period. |
| Psychiatrist and allied services capacity initial | 2017.714721 | The initial number of psychiatrist and allied health services provided per week (i.e., at the start of 2011). Estimated via constrained optimisation. |
| Psychological treatment rate GP services | 0.4818195 | Derived from national data on mental health-related general practitioner services published by the Australian Institute of Health and Welfare (available at: https://www.aihw.gov.au/reports-data/health-welfare-services/mental-health-services/data) |
| Recovery base rate CMHC services | 0.02332282 | Per-service recovery rate derived from data on patient outcomes and numbers of services per patient per year published online by the Australian Institute of Health and Welfare (https://www.aihw.gov.au/reports-data/health-welfare-services/mental-health-services/data) |
| Recovery rate mental health treatment | 0.09525994 (moderate distress), 0.08339287 (high distress) | Per-service recovery rates derived from treatment effectiveness estimates reported in Thase et al. (1997, Arch. Gen. Psychiatry 54, 1009−1015) and data on numbers of services per patient per year published online by the Australian Institute of Health and Welfare (https://www.aihw.gov.au/reports/primary-health-care/medicare-subsidised-gp-allied-health-and-specialis/data). |
| Recovery rate online services | 0.4 (moderate distress), 0.1850746 (high distress) | Derived from Christensen et al. (2004, Br. Med. J. 328, 265) and Cuijpers et al. (2009, Br. J. Gen. Pract., doi: 10.3399/bjgp09X395139) |
| Recovery rate ratio GP services | 1 (moderate distress), 0.4626866 (high distress) | Derived from Cuijpers et al. (2009, Br. J. Gen. Pract., doi: 10.3399/bjgp09X395139) |
| Referral rate CMHC services GP moderate distress | 0.0209817909452 (15-24 years), 0.0203807949659 (25-64 years), 0 (65+ years) | Fraction of patients with moderate psychological distress consulting a general practitioner referred to community mental health care services. Estimated via constrained optimisation. |
| Referral rate online services | 0.047724167 | Derived from national data on mental health-related general practitioner services published by the Australian Institute of Health and Welfare (available at: https://www.aihw.gov.au/reports-data/health-welfare-services/mental-health-services/data) |
| Referral rate psychiatrist and allied services increase per year | 0.008406844 | Estimated via constrained optimisation |
| Referral rate psychiatrist or allied services initial | 0.136682296 | Initial fraction of patients consulting a general practitioner referred to psychiatrist and allied health services (i.e., at the start of 2011). Estimated via constrained optimisation using national data on mental health-related general practitioner services published by the Australian Institute of Health and Welfare (available at: https://www.aihw.gov.au/reports-data/health-welfare-services/mental-health-services/data). |
| Referred to psychiatric hospital base rate | 0.005 | Default value assumes 0.5% of patients with moderate psychological distress receiving psychiatrist and allied health services are referred to psychiatric hospital care |
| Seeking help GP services base rate age array | 1.8365970972 (15-24 years), 3.18348045713 (25-64 years), 0.961793692871 (65+ years) | Per capita rate per year. Estimated via constrained optimisation. |
| Seeking help GP services rate increase per year | 0.005984999 | Estimated via constrained optimisation |
| Seeking help online services rate | 0.09036995 (moderate distress), 0.1985965 (high distress) | Per capita rate per year. Derived from Australian Bureau of Statistics (2012, Information paper. Use of the Kessler psychological distress scale in ABS health surveys, Australia, 2007-08. Cat. no. 4817.0.55.001. Australian Bureau of Statistics, Canberra). |
|  |  |  |
| Suicidal behaviour sector |  |  |
|  |  |  |
| Self-harm hospitalisation rate moderate distress | 0.00124192083336 (15-24 years), 0.000614215197231 (25+ years) | Per capita rate per year. Estimated via constrained optimisation. |
| Self-harm rate ratio high distress | 22.40909 | Derived from Chamberlain et al. (2009, Crisis 30, 39−42) |
| Suicide attempt lethality | 0.0776955 | Estimated via constrained optimisation |
|  |  |  |
| Interventions |  |  |
|  |  |  |
| Acute care effect on self-harm rate | 0.3975155 | Derived from Hvid et al. (2011, Nord. J. Psychiatry 65, 292−298) |
| Acute care services effect duration (weeks) | 2 | Default value assumes community-based acute care reduces the probability of repeat episodes of suicidal ideation or behaviour for a mean of 2 weeks after referral |
| Additional CMHC service contacts per patient (post-attempt care) | 18.24816 | Difference between the mean number of community mental health care service contacts per patient for patients receiving medium to longer term treatment and the mean number of service contacts per patient for all patients. Estimated using data published by the Australian Institute of Health and Welfare (2018, Mental health services − in brief 2018. Cat. no. HSE 211. Australian Institute of Health and Welfare, Canberra). |
| Assertive aftercare effect duration (weeks) | 52.14285714 | Default value assumes assertive aftercare reduces the probability of repeat self-harm for a mean of 52.1 weeks after a suicide attempt |
| Assertive aftercare effect estimate | 0.3975155 | Derived from Hvid et al. (2011, Nord. J. Psychiatry 65, 292−298) |
| Assertive aftercare treatment duration (weeks) | 13.14286 | Default value assumes post-attempt care is provided for a mean of 92 days after a suicide attempt. This is the minimum duration of medium to longer term community-based mental health treatment, as defined in Australian Institute of Health and Welfare (2018, Mental health services − in brief 2018. Cat. no. HSE 211. Australian Institute of Health and Welfare, Canberra) |
| Domestic violence prevention programs effect estimate | 1 | Multiplies domestic violence rates (incidents reported per year) among people aged 15 years and above. The default value corresponds to the baseline case, in which no new policy interventions act to change these rates directly (i.e., business as usual). |
| Early life programs effect estimate | 1 | Multiplies the rates at which children aged 0–14 years at low and moderate risk of developing a mental disorder transition to moderate and high levels of risk. The default value corresponds to the baseline case, in which no new policy interventions act to change these rates directly (i.e., business as usual). |
| ED referral rate acute care services | 0.4815356 | Proportion of patients presenting to an emergency department for suicidal ideation or behaviour admitted to hospital (patients requiring inpatient care are assumed to be admitted via an emergency department). Estimate derived from Perera et al. (2018, Med. J. Aust. 208, 348−353). |
| ED referral rate tech-enabled crisis response | 0.4815356 | Proportion of patients presenting to an emergency department for suicidal ideation or behaviour admitted to hospital (patients requiring inpatient care are assumed to be admitted via an emergency department). Estimate derived from Perera et al. (2018, Med. J. Aust. 208, 348−353). |
| Effect of integrated care on AOD services referral rate | 1.1 | Default value assumes a 10% increase in the per capita rate of referral to alcohol and other drug treatment services |
| Effect of integrated care on disengagement | 0.5204988 | Derived from Badamgarav et al. (2003, Am. J. Psychiatry 160, 2080−2090) |
| Effect of integrated care on employment | 1.1 | Default value assumes a 10% increase in the per capita rate of employment initiation (due to referral to employment services) |
| Effect of integrated care on exiting homelessness rate | 1.1 | Default value assumes a 10% increase in the per capita rate at which homeless patients secure housing (due to referral to homelessness services) |
| Effect of integrated care on recovery rate | 1.177321 | Derived from Woltmann et al. (2012, Am. J. Psychiatry 169, 790−804) |
| Effect of integrated care on referrals to specialised care | 1 (moderate distress), 1.265913 (high distress) | Estimate for patients with high to very high psychological distress derived from Badamgarav et al. (2003, Am. J. Psychiatry 160, 2080−2090). Note that technology-enabled coordinated care is assumed to have no effect on the referral rate for patients with moderate psychological distress. |
| Effect of integrated care on services capacity | 1.1 | Default value assumes a 10% increase in psychiatrist and allied health services capacity |
| Effect of integrated care on substance abuse relapse | 0.8691186 | Derived from Kikkert et al. (2018, J. Subst. Abuse Treat. 95, 35−42) |
| Effect of Sense of Community Index increase on distress | 0.64 | Derived from Handley et al. (2012, Soc. Psychiatry Psychiatr. Epidemiol. 47, 1281−1290) |
| Effect of Sense of Community Index increase on lethality | 0.9637119 | Default value assumes an increase in the Sense of Community Index from its baseline value (9.15) to 12 (the maximum possible value) would reduce suicide attempt lethality by 10% |
| Employment programs effect estimate | 1 | Multiplies the rate at which unemployed young people (aged 15–24 years) secure employment. The default value corresponds to the baseline case, in which no new policy interventions act to change this rate directly (i.e., business as usual). |
| Housing programs effect estimate entering homelessness rate | 1 | Multiplies the age-specific rates at which people in secure housing enter homelessness. The default value corresponds to the baseline case, in which no new policy interventions act to change these rates directly (i.e., business as usual). |
| Maximum acute care services referral rate | 0.7 | Default value assumes that a maximum of 70% of people in suicidal crisis who would usually present to an emergency department will contact community-based acute care services instead |
| Maximum assertive aftercare rate | 1 | Default value assumes that post-attempt care will be provided to all patients hospitalised for a suicide attempt when fully implemented |
| Maximum integrated care rate per service | 0.7 | Default value assumes that technology-enabled coordinated care will be provided in 70% of mental health service contacts when fully implemented |
| Peer support services duration (weeks) | 6 | Default value assumes that patients receive peer support for a mean of 6 weeks after discharge from psychiatric hospital care (see Scanlan et al., 2017, BMC Psychiatry 17, 307) |
| Peer support services effect on disengagement rate | 0.6315789 | Derived from Craig et al. (2004, J. Mental Health 13, 59−69) |
| Peer support services effect on ED presentation rate | 0.5666667 | Derived from Lawn et al. (2008, J. Mental Health 17, 498−508) |
| Peer support services effect on self-harm rate | 0.3975155 | Derived from Hvid et al. (2011, Nord. J. Psychiatry 65, 292−298) |
| Post-discharge ED presentation rate | 5.214286 | Derived from Scanlan et al. (2017, BMC Psychiatry 17, 307) |
| Post-discharge self-harm rate | 0.02314493 | Derived from Olfsen et al. (2016, JAMA Psychiatry 73, 1119−1126) |
| Proportion of ED presentations involving paramedics or police | 0.224 | Derived from Australian Institute of Health and Welfare (2018, Mental health services − in brief 2018. Cat. no. HSE 211. Australian Institute of Health and Welfare, Canberra) |
| Proportion of psychiatric hospital patients receiving peer support target | 1 | Default value assumes that peer support services are provided to all patients discharged from psychiatric hospital care when fully implemented |
| Repeat self-harm rate per year | 0.179 | Derived from Carroll et al. (2014, PLoS ONE 9, e89944) |
| Self-harm re-presentation rate per year | 3.837889 | Derived from Perera et al. (2018, Med. J. Aust. 208, 348−353) |
| Sense of Community Index initial | 9.149557522 | Derived from Handley et al. (2012, Soc. Psychiatry Psychiatr. Epidemiol. 47, 1281−1290) |
| Sense of Community Index target | 9.607035 | Default value corresponds to a 5% increase in the Sense of Community Index (relative to the baseline value, 9.15) |
| Substance abuse prevalence ratio high distress | 2.232085 | Prevalence of substance abuse among people with high to very high psychological distress divided by the prevalence of substance abuse in the total population. Prevalence estimates were derived from Australian Bureau of Statistics (2012, Information paper. Use of the Kessler psychological distress scale in ABS health surveys, Australia, 2007-08. Cat. no. 4817.0.55.001. Australian Bureau of Statistics, Canberra). |
| Suicide-related ED presentation admission rate | 0.4815356 | Derived from Perera et al. (2018, Med. J. Aust. 208, 348−353) |
| Suicide-related ED presentation rate | 0.173505926 | Derived from Perera et al. (2018, Med. J. Aust. 208, 348−353) |
| Tech-enabled crisis response effect duration (weeks) | 2 | Default value assumes technology-enabled crisis response care reduces the probability of repeat episodes of suicidal ideation or behaviour for a mean of 2 weeks |
| Tech-enabled crisis response effect on self-harm rate | 0.3975155 | Derived from Hvid et al. (2011, Nord. J. Psychiatry 65, 292−298) |
| Unemployment increase effect | 1 | Multiplies the age-specific rates at which employed people (aged 15 years or more) become unemployed. The default value corresponds to the baseline case (i.e., business as usual). |
| Unemployment rate ratio high distress | 2.300218 | Unemployment rate among people with high to very high psychological distress divided by the total unemployment rate. Unemployment rate estimates were derived from Australian Bureau of Statistics (2012, Information paper. Use of the Kessler psychological distress scale in ABS health surveys, Australia, 2007-08. Cat. no. 4817.0.55.001. Australian Bureau of Statistics, Canberra). |
| Years to implement domestic violence prevention programs | 1 | Default value assumes it will take 1 year to fully implement domestic violence programs |
| Years to implement early life programs | 1 | Default value assumes it will take 1 year to fully implement early life programs |
| Years to implement employment programs | 1 | Default value assumes it will take 1 year to fully implement youth employment programs |
| Years to implement housing programs | 1 | Default value assumes it will take 1 year to fully implement housing programs |
| Years to increase unemployment | 1 | Default value assumes it will take 1 year for increases in unemployment to be fully realised |
| Years to reach maximum acute care services rate | 2 | Default value assumes it will take 2 years to reach the maximum acute care services referral rate |
| Years to reach Sense of Community Index target | 10 | Default value assumes it will take 10 years to reach the Sense of Community Index target |
| Years to scale up assertive aftercare | 2 | Default value assumes it will take 2 years to fully implement an post-attempt care program |
| Years to scale up integrated care | 2 | Default value assumes it will take 2 years to fully implement technology-enabled coordinated care |
| Years to scale up peer support services | 2 | Default value assumes it will take 2 years to fully implement a peer support program for patients discharged from psychiatric hospital care |
| Years to scale up tech-enabled crisis response services | 1 | Default value assumes it will take 1 year to fully implement technology-enabled crisis response services |
|  |  |  |
